# Development and Initial Validation of a Multidimensional Instrument for Assessing Determinants of HPV Vaccine Readiness and Uptake Among Caregivers in Pakistan: A PLS-SEM Approach

**DOI:** 10.3390/vaccines14090761

**Published:** 2026-08-31

**Authors:** Khola Noreen, Samina Naeem Khalid, Saba Maryam, Mehreen Noor, Abdul Momin Rizwan Ahmad

**Affiliations:** 1 Department of Community Medicine & Public Health, Rawalpindi Medical University, Rawalpindi 46000, Pakistan; khauladr66@gmail.com (K.N.); saba.maryam@rmur.edu.pk (S.M.); mehreen.noor@rmur.edu.pk (M.N.); 2 School of Public Health, Health Services Academy, Islamabad 44000, Pakistan; drsamina@hsa.edu.pk; 3 Department of Human Nutrition and Dietetics, NUST School of Health Sciences, National University of Sciences & Technology (NUST), Islamabad 44000, Pakistan; 4 Department of Health Sciences, University of York, York YO10 5DD, UK

**Keywords:** HPV vaccine, instrument validation, PLS-SEM, Pakistan, vaccination uptake, psychometrics, public health

## Abstract

**Background**: HPV vaccination coverage in Pakistan is extremely low, and there are complex psychosocial, cultural, and logistical barriers that currently impede vaccinations. There is no validated measurement tool that is appropriate to the context to measure these determinants systematically. **Objective**: To develop and validate a multidimensional instrument measuring important factors associated with HPV vaccine uptake and readiness in Pakistan through partial least squares structural equation modelling (PLS-SEM). **Methods**: A cross-sectional survey was administered to caregivers of girls aged 9–16 years, recruited via purposive/convenience sampling from schools, EPI sites, and household outreach in three Punjab/ICT districts (Rawalpindi, Islamabad, Bahawalpur) during September–November 2025, coinciding with Pakistan’s national HPV vaccination campaign (700 initial responses; 639 retained after screening). The instrument comprised 75 initial items across six constructs: thinking and feeling, social processes, motivation, practical issues, cultural integration, and the vaccination uptake composite index (a composite index of actual vaccination receipt, intention, perceived importance, and perceived access). The measurement model was evaluated for indicator/construct reliability, convergent validity (AVE), and discriminant validity (HTMT, Fornell–Larcker, cross-loadings); the structural model tested direct effects, mediation via motivation, and moderation by practical issues and cultural integration. **Results**: The number of indicators after model refinement was 27 in six constructs. All outer loadings exceeded 0.70 (range: 0.765–0.939). Cronbach’s α ranged from 0.854 to 0.928, composite reliability from 0.912 to 0.945, and AVE from 0.710 to 0.850. All the HTMT ratios were less than 0.90; Fornell–Larcker and cross-loadings criteria supported discriminant validity. The relationships between thinking and feeling (β = 0.098, *p* < 0.001) and social processes (β = 0.155, *p* < 0.001) with vaccination uptake were significantly mediated by motivation. Social and cognitive pathways were moderated by cultural integration. The model accounted for 65.8% of the variance in motivation and 59.4% in uptake of vaccination. **Conclusions**: The instrument was found to have good psychometric properties and to be suitable suitable for evaluating the determinants of HPV vaccination in conservative, resource-constrained countries such as Pakistan. Findings have direct implications for targeted public health interventions to improve HPV vaccine readiness and uptake.

## 1. Introduction

Human papillomavirus (HPV) infection is the most common sexually transmitted viral infection worldwide and is the primary cause of cervical cancer. Cervical cancer remains a major public health concern, particularly in low- and middle-income countries (LMICs), where approximately 90% of cervical cancer deaths occur because of limited access to HPV vaccination, screening, and timely treatment [1,2,3].

HPV vaccination rates in Pakistan are very low, and cervical cancer (CC) is the second most prevalent cancer in women of reproductive age (15–44 years) [2,3]. Although considerable achievements have been made in school-based vaccination programmes in high-income countries, Pakistan is still facing the challenge that the country does not have an HPV immunisation policy, and few private health facilities can provide the HPV vaccine [4]. Religious, cultural, and social beliefs are deeply rooted causes of vaccine hesitancy in Pakistan, beyond structural barriers [5,6,7,8]. Low health literacy, religious leaders’ influence on community health behaviour, mistrust in the Western-manufactured vaccines, and misinformation suggesting that HPV vaccination could lead to promiscuity all play a role in suboptimal vaccine readiness and uptake [5,6,7,8]. In addition, the high costs of vaccines, a lack of capacity for healthcare providers, and low-reach programmes are major obstacles, both in urban and rural contexts [6]. There has been substantial progress in HPV school-based vaccination programmes in high-income countries. In Pakistan, HPV vaccine availability had been restricted to private health facilities and no national HPV immunisation policy had been available until recently [4]. The landscape has shifted dramatically with the initiation of Pakistan’s first national HPV vaccination programme on 15 September 2025, spearheaded by the Federal Directorate of Immunization, in collaboration with Gavi, the Vaccine Alliance, UNICEF, and the World Health Organization. The first phase of the campaign targeted girls only, aged 9–14 years, across Punjab, Sindh, Islamabad Capital Territory, and Pakistan-administered Kashmir; the vaccine was provided free of charge through fixed health facilities, schools, and outreach/mobile teams, with a target of reaching at least 90% of an estimated 13 million eligible girls. Subsequent phases are planned to extend coverage to Khyber Pakhtunkhwa, Balochistan, and Gilgit-Baltistan, alongside integration of the vaccine into routine immunisation for 9-year-old girls. With technical support from the WHO and allied partners, the Federal Directorate of Immunization and provincial health sectors recently executed Phase I of an HPV vaccination campaign against cervical cancer. The drive focused on 9- to 14-year-old girls, both in and out of the schooling system, across Islamabad, AJK, Sindh, and Punjab between 15–27 September.

To ensure maximum reach, organisers added a three-day catch-up window from 29 September to 1 October 2025, bringing the total campaign length to 15 days. Over this timeframe, 9.7 million girls were administered a single dose of the Cecolin vaccine. The bulk of these immunisations occurred in Punjab (6.87 million) and Sindh (2.67 million), while AJK and Islamabad vaccinated 116.7 thousand and roughly 56 thousand girls, respectively.

Ultimately, the initiative secured a 75% national vaccination rate. Provincial and regional coverage varied, topped by Punjab at 81%. Sindh followed with a 66% coverage rate, while AJK and Islamabad recorded 43% and 38% coverage, respectively [9,10].

Although there has been increasing focus on HPV vaccination in the global health literature, there is a lack of validated tools that can measure the multidimensional determinants of HPV vaccine readiness and uptake in Pakistan’s sociocultural setting [6]. Tools built in the West are often not sensitive to the distinct cognitive, emotional, social, and structural factors found in conservative, low-resource settings. Moreover there is a lack of tools validated tools that have been assessed using robust statistical techniques, including structural equation modelling, which can address both construct complexity and non-normal data distributions [11,12].

Several behavioural theories and validated survey instruments have been developed to explain determinants of HPV vaccination uptake. The health belief model(HBM) has been widely applied to examine perceived susceptibility, perceived severity, perceived benefits, perceived barriers, cues to action, and self-efficacy associated with HPV vaccination decisions. Although HBM has been extensively used, yet it has several shortcomings in the sociocultural context of Pakistan. It overemphasises individual cognition and can be useful in understanding parental risk perceptions of HPV but has a limited role in determining vaccine behaviour in the complex sociocultural context of Pakistan, neglecting patriarchal influence, gender roles, and religious and sociocultural barriers influencing vaccination uptake [13].

TPB has widely been used in the vaccination research literature to investigate parental decision-making and intention formation especially about new or controversial vaccines [11]. TPB, therefore, is an improvement over its predecessors because it recognises that intention might not always lead to action when behavioural control is limited. Nevertheless, TPB possesses significant weaknesses in motivating vaccination among culturally complex situations. The model presupposes rational decision and overly simplifies emotional, moral, and religious aspects that are vital in vaccine acceptance, especially regarding vaccines related to sexual health, including HPV. In addition, TPB fails to adequately explain the role of power relations, gendered roles, or institutional trust, which plays a decisive role in influencing vaccination in Pakistan [14].

The COM-B model, a systematic and integrative conceptual model of behaviour change, was created by Michie et al. that hypothesises that behaviour results from the interaction between three fundamental elements: capability (C), opportunity (O), and motivation (M). Over time, this framework has emerged with growing prominence in the field of vaccination research because of its usefulness in identifying modifiable barriers on the basis of which interventions can be designed. In spite of these facts, the COM-B model has weaknesses in contexts with cultural complexities. Although it adequately reflects psychological, social, and environmental factors, it fails to explicitly include the strong and deeply entrenched cultural norms, moral values, and religious beliefs that influence the vaccination decision in Pakistan. Consequently, the model needs contextualization to achieve cultural sensitivity and relevance [15].

By contrast, the WHO-BeSD framework provides both a comprehensive and vaccination-specific model comprised of cognitive, social, motivational, and practical factors that influence vaccine uptake [16]. The framework is particularly pertinent to the multifaceted influences on HPV decisions in Pakistan, as it explicitly encompasses the domains touching on thinking and feeling, social processes, motivation, and practicality [17]. Furthermore, the WHO-BeSD framework is modelled to assist in both measuring and policy-focused decision-making, which makes it highly beneficial in informing evidence-based vaccine introduction practices. However, as a globally developed concept, its successful implementation in the Pakistani context requires systematic cultural adaptation and careful psychometric validation [18].

This study addresses this need by developing and psychometrically validating a new multidimensional instrument that operationalises six theoretically grounded constructs: thinking and feeling; social processes; motivation; practical issues; cultural integration; and vaccination uptake. Partial least squares structural equation modelling (PLS-SEM) was used for instrument development and validation; the rationale for this analytical choice is described in the Statistical Analysis subsection of the Methods section [19].

The specific aim of this study was to; (1) create an item pool that captures a broad range of factors that influence HPV vaccination, relevant to the Pakistani population; (2) evaluate the psychometric properties of the measurement model (reliability, convergent, and discriminant validity); (3) test the structural model (direct, indirect, and moderating effects); and (4) to develop a rigorously validated measure of these factors for future epidemiological and public health research in Pakistan.

## 2. Materials and Methods

### 2.1. Study Design and Setting

A quantitative cross-sectional study was carried out in northern Punjab (Rawalpindi District) Islamabad Capital Territory(ICT) and southern Punjab (Bahawalpur District). The selected districts were strategically selected to represent cultural and health service disparities in Punjab. Islamabad is the capital territory, and Rawalpindi comprises urban areas with greater availability of health services and immunisation programmes. Bahawalpur is a more rural and traditional area where cultural norms and access to services present more challenges. This was intended not only to obtain a representative sample but also to ensure the evaluation of the impact of human behaviour, society, and culture in different contexts on HPV vaccination uptake. This study adhered to the ethical guidelines, and ethical approval was obtained from the Graduate Research Management Council (GRMC) of Health Services Academy under reference no. F. 00015/HSA/PhD-2022 dated 24 February 2025. Participant anonymity and data confidentiality were maintained, and written informed consent was also obtained from all study participants.

Data collection for the present study was conducted between September and November 2025, during the national vaccination campaign. As data collection coincided with an active, highly publicised national campaign, respondents’ awareness, perceived access to vaccination services, and reported vaccination-related behaviour may have been directly influenced by ongoing campaign messaging and service availability; this is an important contextual factor for interpreting the study’s findings and is addressed further in the limitations.

### 2.2. Study Population and Sampling

The target group consisted of adults (mainly mothers and caregivers) participating in the decision-making process about girls’ health and vaccination (aged 9–16 years). The respondents were selected using non-probability convenience sampling to achieve a representative cross section of attitudes towards HPV vaccination across educational and socioeconomic groups.

As the study specifically focused on adolescents’ caregivers as active decision-makers for adolescent girls, elderly caregivers (aged over 65 years) were not included in the study as their decision-making might vary in Pakistan depending on cultural background, and their inclusion might skew the data. Additionally, individuals were excluded if, despite being mothers or caregivers, they delegated all health-related decisions or were not actively involved in such decisions, as their insights into the determinants of HPV readiness and uptake would be limited. Exclusion also applied to individuals who did not speak or understand Urdu, given that the instrument was translated into Urdu, and participants unable to comprehend the questionnaire accurately would provide unreliable data. Furthermore, individuals with severe cognitive impairments or mental health conditions that would prevent them from understanding the questionnaire or providing informed consent were excluded. Finally, healthcare professionals directly involved in HPV vaccination programmes were excluded, as their professional knowledge and potential biases might significantly differ from the general caregiver population, thereby influencing their responses in a way that would not reflect typical community determinants.

These exclusion criteria collectively limited the generalizability of the findings to a specific demographic, particularly younger and middle-aged, actively involved, Urdu-speaking lay caregivers without severe cognitive impairments. The results may therefore not be entirely reflective of the wide cultural, linguistic, and decision-making diversity of the wider Pakistani population.

Nevertheless, adolescent girls aged 9–14 years were selected as the target population because the primary objective of this study was to support the introduction of HPV vaccination in Pakistan. This is consistent with the ongoing cervical cancer prevention campaign in the country that focuses on the vaccination of adolescent girls. This is the phased programme implementation strategy used by many LMICs, where girls-only vaccination is targeted in order to prioritise finances, vaccine supply, and programme delivery. Importantly, boys were not conceptually excluded from being included in the study; however, boys were not targeted as the primary study population as they were not part of the current national HPV vaccination strategy.

The study enrolled participants through a multi-pronged recruitment strategy that included facility-based approaches at schools and fixed Expanded Programme on Immunization (EPI) sites, as well as household-based recruitment via community outreach activities. Eligible respondents were approached and screened at these various recruitment sites. The enrolment period for the study was from September to November 2025. The questionnaire was administered in an interviewer-administered format.

Before the main data collection period, 50 subjects were given the instrument to pre-test to determine the face validity and comprehensibility. Cronbach’s alpha values for some pre-test items showed acceptable reliability, and slight modifications were made to the wording of the items based on the participants’ feedback.

Data was collected using a structured electronic questionnaire administered through the KoboToolbox platform. The questionnaire was designed using the culturally adapted WHO Behavioural and Social Drivers (BeSD) framework and put together into the secure, standardised, and real-time data collection platform KoboToolbox. The survey was completed by trained data collectors using tablets and smartphones after participants had been identified and had provided written informed consent. To limit missing data and data entry errors, the electronic platform was programmed to include range validation checks, required response fields, and skip logic. The completed questionnaires were submitted to a secure KoboToolbox server every day and then exported to Microsoft Excel and IBM SPSS Statistics version 27 where the data was cleaned and coded, and preliminary analyses were performed. The initial 700 responses were subjected to extensive data screening, and the samples that contained ‘outliers’ or other irregularities were discarded from the analysis, leaving 639 responses for analysis. The finalised data set was then exported to SmartPLS software (Version 4.0) for partial least squares structural equation modelling (PLS-SEM): the measurement model (reliability and validity) and the structural model (path coefficients, explanatory power, predictive relevance, and hypothesis testing).

### 2.3. Instrument Development

The instrument was systematically developed based on a review of the HPV vaccination literature and theoretical models of health behaviour change: the health belief model, theory of planned behaviour, and social cognitive theory.

**Phase 1: Item Generation and Cross-Cultural Adaptation**

The first item pool consisted of 75 items, which were developed in a systematic manner through a comprehensive search of the literature on HPV vaccination along with HPV-specific health behaviour change theories such as the health belief model, the theory of planned behaviour and social cognitive theory. To deepen the theoretical framework and provide context, deductive and inductive coding were used to analyse data from preliminary qualitative research and to generate themes following Braun and Clarke’s six-step analysis. Existing WHO Behavioural and Social Drivers (BeSD) domains (thinking and feeling, social processes, motivation, and practical issues) were matched using a deductive approach [16]. Crucially, inductive analysis revealed a new, context-specific domain: cultural integration. Themes and subthemes were meticulously mapped to these specific BeSD constructs and the emergent cultural integration domain, illustrated with participant quotations and rationales. This process informed the generation of items across these six constructs.

To ensure cultural and linguistic appropriateness for the Pakistani context, the instrument underwent a meticulous cross-cultural adaptation process, guided by the emic–etic paradigm and Beaton et al.’s [20] internationally accepted guidelines. This involved a forward–backward translation procedure of the initial 75 items into Urdu by independent bilingual experts, followed by expert synthesis to achieve semantic and conceptual equivalence. The tool was subsequently translated into Urdu by a bilingual expert to ensure linguistic appropriateness [17]. The complete wording of the initial 75 items in English and Urdu is available in Supplementary File S1.

**Phase 2: Content Validity**: The instrument’s validation followed a rigorous two-phase method. This approach integrated expert content validation and cognitive interviews to ensure both linguistic and cultural appropriateness for the Pakistani context.

**Phase 2a: The Delphi Method:**

A two-round Delphi method was applied for the systematic assessment of content validity. Each item was judged on a 4-point Likert scale of relevance and clarity by ten experts, including public health experts, gynaecologists, psychologists, an anthropologist, a behaviour scientist, and medical educationists. Item-level CVI scores of ≥0.80 were predefined as having reached consensus for the retention of each item. In Round 1, two items were dropped for low I-CVI scores or lack of conceptual fit during this expert review, and in Round 2, three more items were dropped because of their I-CVI scores or lack of conceptual fit. A total of 28 items were accepted without changes, while 42 items were revised to make them clearer and culturally more relevant after receiving expert feedback. The final 70-item BeSD-HPV tool had a strong scale-level content validity index (S-CVI) score of 0.96.

**Phase 2b: Response Process Validity through Cognitive Interviews**

A total of 30 caregivers of adolescent girls (aged 9–16 years) participated in these interviews. The “think-aloud” technique coupled with selective probing was used to determine item comprehension, cultural appropriateness, and the consistency of the responses as perceived by the target population. Based on the rich qualitative feedback gathered, seventeen items were identified as having minor or major comprehension issues. These items were subsequently revised by the authors to further enhance clarity and cultural relevance and to ensure that the instrument accurately captured the intended constructs within the Pakistani sociocultural context.

This comprehensive development and validation process is fully described in published work [18].

The current study aimed to evaluate the psychometric properties of the adapted BeSD-HPV tool, with purpose of examining relationships among behaviour and social determinants of HPV vaccine readiness and uptake to validate a multidimensional structural model using partial least squares structural equation modelling (PLS-SEM).To achieve this objective, it was methodologically essential to specify an endogenous outcome construct representing HPV vaccination uptake. Accordingly, a latent construct “vaccination readiness and uptake Composite Index” comprising of five items was incorporated into measurement model. These items were not newly developed rather derived from original WHO BeSD framework. In current study, this construct was operationalised as independent endogenous construct to represent outcome variable in structural equation model. This enabled the empirical assessment of the direct, indirect and moderating effects the explanatory constructs. Therefore, by incorporating “Vaccination Readiness and Uptake Composit Index” as a dependent variable, the proposed framework provides a comprehensive understanding of behavioural and social drivers collectively influencing HPV vaccination uptake in the Pakistani context, while maintaining the conceptual integrity of the original WHO BeSD framework.

Six theoretically different constructs were captured in items:

1. **Thinking and feeling** (19 initial items; D1_1–D1_19) determines the thinking and feeling component, such as the level of trust in HPV vaccines, perceived protection, religious acceptability, and attitude towards vaccine safety.
2. **Social processes** (13 items; D2_1–D2_13) assesses social influence, family and peer pressure, community norms, healthcare worker advice, and cultural and religious authority.
3. **Motivation** (8 items; D3_1–D3_8) evaluates internal readiness and willingness to vaccinate, perceived disease risk, and past immunisation experience.
4. **Practical issues** (18 items; D4_1–D4_18) measures access to information, the availability of HPV vaccines, healthcare provider–patient communication, and service-level readiness.
5. **Cultural integration** (12 items; D5_1–D5_12) explores intra-household decision-making processes, vaccination decision-making processes at the household level, and the cultural salience and visibility of HPV and the roles of men in HPV vaccination decisions.
6. **Vaccination readiness and uptake composite index** (5 items; D6_1–D6_5) measures vaccination behaviour as an outcome construct. The original five-item domain was intended to capture the ultimate status of HPV vaccination and factors reflecting readiness to vaccinate. Four indicators were retained following the item assessment: actual HPV vaccination receipt (D6_2), caregiver intention/willingness to vaccinate the eligible girl (D6_3), perceived importance of HPV vaccination (D6_4), and perceived ease of access to HPV vaccination (D6_5). These indicators represent conceptually distinct but related dimensions of the vaccination continuum, ranging from actual vaccination behaviour to intention, perceived value, and practical accessibility. Therefore, they should not be interpreted as interchangeable reflective manifestations of a single underlying psychological construct. Rather, they are best conceptualised as components contributing to a composite measure of vaccination readiness and uptake, with each indicator capturing a distinct aspect of the pathway from willingness and perceived feasibility to actual vaccination.

The items were measured on a 5-point Likert scale (1 = strongly disagree to 5 = strongly agree). The instrument was translated into Urdu language, then back translated by an independent bilingual expert and reconciled by the research team and semantic equivalency was achieved.

### 2.4. Data Screening 

Data were carefully examined for quality and analytic appropriateness before being analysed. The screening process consisted of:

(1) Out-of-range assessment;
(2) Out-of-range data detection and treatment;
(3) Univariate out-of-range data detection (Z-score, threshold: |Z| > +3.29);
(4) Multivariate out-of-range data detection (Mahalanobis distance, *p* < 0.001);
(5) Normality assessment (Kolmogorov–Smirnov (KS) test, Shapiro–Wilk (SW) test, supplemented by skewness and kurtosis statistics).

There were no missing data or out-of-range values found. After the removal of outliers, the analytic sample consisted of N = 639 participants. Formal significance tests for non-normality were performed; however, PLS-SEM can be used under non-normality conditions without the need for multivariate normality. Common method variance (CMV) was measured using Harman’s single-factor test, and the results revealed that there was no single factor accounting for a majority of the variance, thus indicating that CMV did not pose a major threat.

### 2.5. Item Reduction Procedure

Item reduction from the initial 75-item pool to the final 27-item measurement model followed an iterative, criterion-based procedure conducted within the PLS-SEM measurement model: (1) estimation of the full measurement model with all items assigned to their hypothesised construct; (2) sequential removal of the single lowest-loading indicator falling below the 0.70 threshold, followed by model re-estimation; (3) repetition of this process until all retained indicator loadings exceeded 0.70; (4) review of the excluded item’s content relative to the construct’s theoretical definition at each removal step to avoid disproportionately narrowing any construct’s conceptual domain. Items were evaluated against dual statistical and conceptual criteria; the complete item reduction rationale for all 75 preliminary items is provided in Supplementary File S2.

### 2.6. Statistical Analysis

The software used for all the analyses was SmartPLS 4 software. Partial least squares structural equation modelling (PLS-SEM) was selected as the analytical approach because it is well suited to exploratory modelling of newly developed psychometric instruments, performs robustly with data that may not be multivariate normal, and is appropriate for the sample size available in this study [13,14,15,19]. Unlike covariance-based SEM, PLS-SEM maximises explained variance in endogenous constructs and imposes fewer distributional assumptions, which suited the combined instrument-development and structural-testing objectives of this study.

The specification of the proposed structure model was guided by the theoretical underpinnings of WHO Behavioural and Social Drivers (BeSD) framework supported by complementary behaviour theories, including the theory of planned behaviour (TPB), the health belief model (HBM), and the social ecological model (SEM) [21]. The mediation and moderation analyses were guided by established behaviour change theories. Motivation was conceptualised as the proximal determinant of behavioural intention and action, consistent with the health belief model (HBM) and the theory of planned behaviour (TPB), which propose that individual beliefs, attitudes, and intentions directly influence health-related behaviours. Accordingly, motivation was examined as a mediator of the relationship between the independent variables and the study outcome. Practical issues (e.g., accessibility, time, and financial constraints) and cultural integration were considered contextual factors that may strengthen or weaken this relationship, in line with social cognitive theory, which emphasises the interaction between personal, environmental, and behavioural determinants. Therefore, these variables were evaluated as moderators. Alternative analytical specifications, including serial mediation and additional moderator combinations, were beyond the scope of the present study and were not examined. This is acknowledged as a limitation and may be explored in future research.

The analytical process was conducted in two stages using the PLS-SEM approach: measurement model and structural model.

### 2.7. Measurement Model Assessment

To evaluate the measurement model, indicator reliability was determined via outer loadings, with values of ≥0.70 deemed satisfactory [13]. The absence of severe multicollinearity was confirmed using the variance inflation factor (VIF), requiring scores of <5.0. Internal consistency was validated through three metrics: Cronbach’s alpha (≥0.70), Rho_A (≥0.70), and composite reliability (CR) falling within the optimal 0.70 to 0.95 range. Average variance extracted (AVE) values of ≥0.50 were used to confirm convergent validity. Furthermore, discriminant validity was verified using three distinct methods: ensuring the Heterotrait–Monotrait (HTMT) ratio remained below 0.90; applying the Fornell–Larcker criterion (FLC), which dictates that the square root of a construct’s AVE must surpass its correlations with other constructs; and examining cross-loadings. Finally, model fit was judged satisfactory if the standardised root mean square residual (SRMR) was <0.08 and the normed fit index (NFI) achieved a level of ≥0.90.

### 2.8. Structural Model Evaluation

Assessment of the structural path model relied on a bootstrapping procedure utilizing 5000 resamples to compute bias-corrected and accelerated (BCa) confidence intervals. The significance of direct relationships was interpreted by examining *p*-values, t-statistics, and standardised path coefficients (β). The magnitude of these relationships was gauged using Cohen’s f^2^, categorised into small (0.02), medium (0.15), and large (0.35) effect sizes. The mediating role of motivation between the antecedent constructs (thinking and feeling; social processes) and the vaccination readiness and uptake composite index was examined through specific indirect effects. Mediation was evaluated using specific indirect effects, with statistical significance established via 5000 bootstrapped subsamples and 95% bias-corrected and accelerated (BCa) confidence intervals excluding zero. Moderation effects of practical issues (on motivation → the vaccination readiness and uptake composite index) and cultural integration (on thinking and feeling → motivation; social processes → motivation) were tested using product-indicator interaction terms. Predictive power was assessed via the coefficient of determination (R^2^), and predictive relevance was evaluated using the blindfolding-based Q^2^ statistic (weak: 0.02; moderate: 0.15; strong: 0.35).

## 3. Results

### 3.1. Pre-Testing

The research instrument was pre-tested with 50 participants. Table 1 presents the results of the Cronbach alpha for the instrument. Reliability values of 0.70 or higher were acceptable, 0.80 or higher were good, and 0.90 or higher were excellent. Values less than 0.70 were frequently considered as “marginally acceptable” particularly when conducting pre-testing or exploratory research [22].

Results reveal good reliability for most constructs. Vaccination readiness and uptake composite index also had an acceptable alpha coefficient of 0.794. The alpha coefficient for thinking and feeling was 0.697, which is slightly lower than the standard of 0.70, but it can be regarded as marginally acceptable at the pre-testing stage. Hence, the instrument had good internal consistency and was appropriate for main-study data collection.

### 3.2. Data Preparation

The data was screened for data entry errors, missing values, outliers, normality, and common method variance prior to the main analysis. A total of 700 responses were collected, while 639 responses were retained for the main-study analysis.

#### 3.2.1. Out-of-Range Values

The out-of-range values were checked in the dataset to detect any invalid responses that fell outside the range of the responses. No values were detected outside of the range and therefore the 700 responses collected were found valid.

#### 3.2.2. Missing Value Analysis

A missing value analysis was performed to explore the data set for missing values. The results revealed that there were no missing data in the collected data. All 700 responses were thus retained after the missing value screening.

#### 3.2.3. Univariate Outliers

Standardised Z-score values for individual items were estimated, and a total of 55 univariate outliers were detected and removed from the dataset based on the recommended threshold for Z-scores exceeding ±3.29 [23]. After removing these cases, 645 valid responses remained for further screening.

#### 3.2.4. Multivariate Outliers

The remaining 645 responses were further screened for multivariate outliers. The Mahalanobis distance (D^2^) was estimated for multivariate outlier detection, with a recommended threshold of D^2^ < 0.001 [23]. Six multivariate outliers were detected and removed. After this step, 639 responses were retained for the final main-study analysis.

#### 3.2.5. Normality Assessment

The normality of the data was tested by using skewness, kurtosis, Kolmogorov–Smirnov (KS), and Shapiro–Wilk (SW) statistics The skewness values were between −0.957 and 0.134, and the kurtosis values were between −1.231 and 1.944. These values fall within the commonly accepted range of ±2.00 [24]. This indicates that there were no serious problems of non-normality in the data. However, the results indicated that all the items had significant values in both the Kolmogorov–Smirnov (KS) and Shapiro–Wilk (SW) tests, with *p*-values reported as <0.001 [25]. This means that there was a significant departure from the normal distribution. Hence, the data had a non-normal distribution.

#### 3.2.6. Common Method Variance

The Harman [26] single-factor test was used to check common method variance, and the results revealed a single-factor variance of 29.665%. Since this value is below the recommended threshold of 50% [27], common method variance was not considered a serious issue in the study.

Full collinearity VIF analysis was also conducted as a supplementary test for common method bias. All inner model VIF values were below the recommended threshold of 3.3 [13], ranging from 1.015 (practical issues × motivation) to 3.012 (cultural integration × thinking and feeling). This confirms that common method variance does not substantially distort the structural estimates and corroborates the initial Harman single-factor test result. 

### 3.3. Demographic Profile of the Respondents

Table 2 shows the demographic characteristics of the respondents studied. As the survey was conducted during and immediately after the first phase of Pakistan’s national HPV vaccination campaign (September–November 2025), out of total 639 participants 62.5% (400) reported that they have received at least one dose of HPV vaccine during campaign. we now report the number and proportion of eligible girls who had received at least one dose of the HPV vaccine at the time of data collection.

### 3.4. Sample Characteristics

The final sample consisted of 639 participants. Most of them were female, i.e., mothers who had a primary role in decision-making regarding healthcare. The participants had a diverse education status and household income brackets. The majority of respondents indicated that the girls in their household aged 9–16 years had received at least one vaccination dose, indicating a high level of immunisation, though the HPV vaccination rates within this cohort were variable.

### 3.5. Descriptive Statistics

Table 3 presents descriptive statistics for all items for all responses across six constructs.

For the thinking and feeling construct (D1; 19 items), mean scores ranged from 2.79 (D1_15) to 3.97 (D1_18), with standard deviations between 0.698 and 1.235. The social processes construct (D2; 13 items) yielded mean scores of 3.05–4.11 and standard deviations of 0.631–1.148. Motivation items (D3; 8 items) had means ranging from 2.98 to 3.95 and standard deviations of 0.671–1.016. Practical issues (D4; 18 items) demonstrated the widest range of means (2.74–4.36) and standard deviations (0.551–1.314). Cultural integration items (D5; 12 items) had mean scores of (3.18–4.00), with standard deviations of 0.618–1.134. Vaccination readiness and the uptake composite index (D6; 5 items) showed a narrower range of means (3.23–3.48) and standard deviations (0.863–1.001), consistent with the outcome-based nature of this construct.

All items showed significant departure from normality based on KS and SW tests (all *p* < 0.001). Skewness values were generally modest (range: −0.957 to 0.134), suggesting acceptable distributional shape for the PLS-SEM analysis, which does not require multivariate normality. Kurtosis values were also within acceptable ranges for the majority of items.

### 3.6. Measurement Model

**Figure 1:**
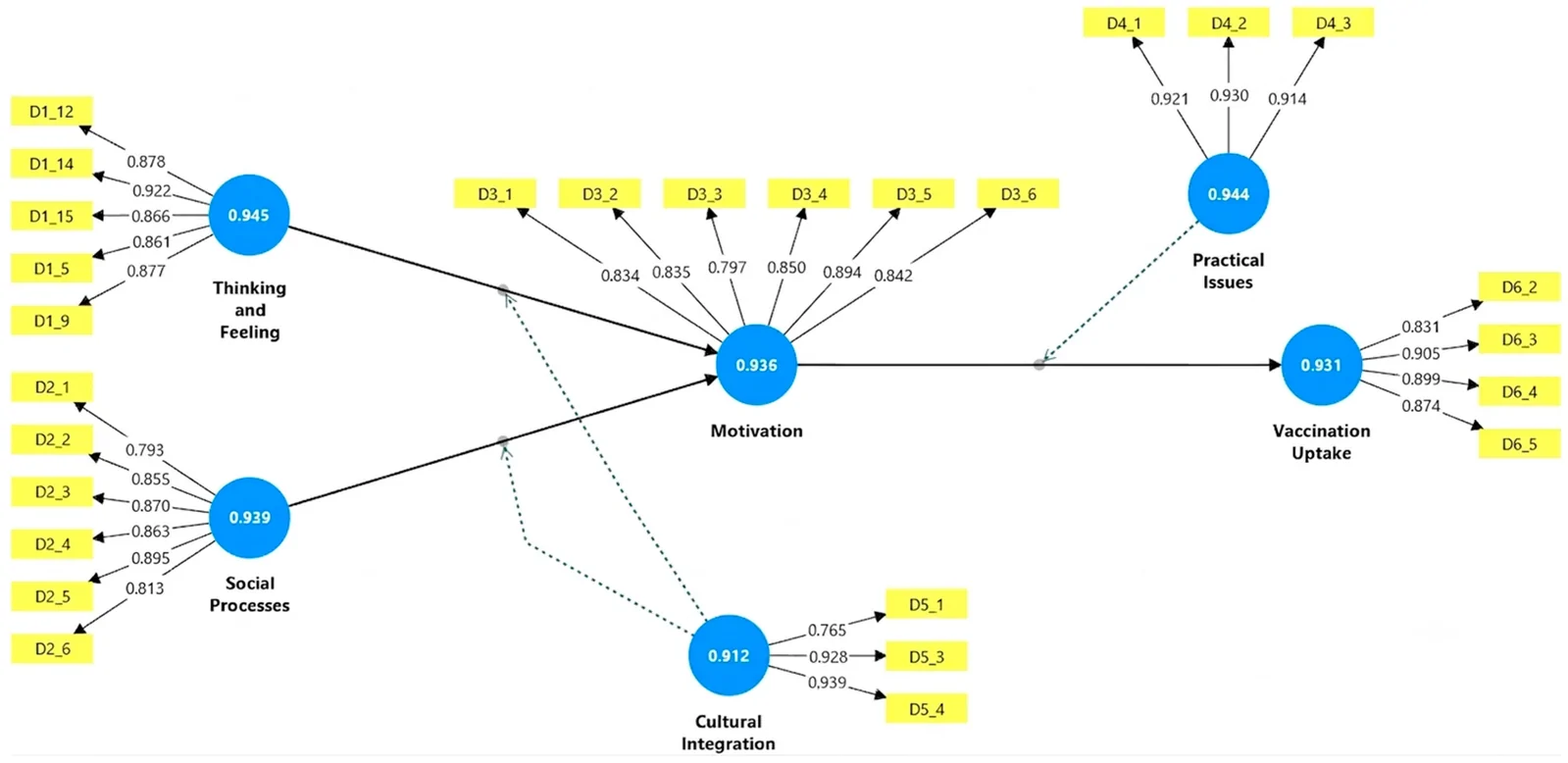
PLS algorithm using SmartPLS. Latent constructs (blue circles) and their observed indicators (yellow rectangles) are shown with standardized factor loadings. Solid paths between constructs indicate direct relationships, while dashed arrows highlight the moderating effects of Cultural Integration and Practical Issues on the main pathways.

A measurement model evaluation was carried out to ensure that all constructs exhibited the necessary reliability and validity before examining structural relationships. The main objective was to confirm that the scales met the established quality criteria and provided a solid foundation for subsequent analysis in the PLS-SEM framework [19,28] (Figure 1).

#### 3.6.1. Indicator Reliability

After iterative model refinement, 27 indicators were selected from a pool of 75 items. They were deleted where outer loadings fell below 0.70 or where conceptual breadth undermined construct parsimony. The exact wording of the final 27 retained items is provided in Supplementary File S3.

Table 4 shows the outer loadings, probability values, and VIF coefficients for all retained indicators.

The outer loadings for all of the retained indicators are acceptable and in excess of the recommended loading of 0.70 [13], ranging from 0.765 to 0.939, with all indicators being statistically significant at 1%. The variance inflation factor (VIF) values are between 1.578 and 4.479, which is less than the acceptable value of 5 [13], indicating that there is no multicollinearity problem between the indicators.

Thinking and feeling retained five indicators (i.e., D1_12, D1_14, D1_15, D1_5, and D1_9), with loadings ranging between 0.861 and 0.922 and significant *p*-values. These indicators were retained because they reflect trust in HPV vaccine, religious acceptability, acceptance in a conservative society, willingness even when family history of cancer is low, and perceived protection from serious illness. These are retained indicators that show the belief-based and emotional aspect of vaccine acceptance in Pakistan. Additional indicators related to safety, knowledge, side effects, natural immunity, social media, and policy acceptance were excluded, as they represented broader themes, rather than the strongest core dimension.

Six indicators (D2_1 to D2_6) were retained for social processes with loadings between 0.793 and 0.895. The retained indicators were social pressure, family influence, health worker advice, permission, community rumours, and religious influence. These indicators were retained because they constituted a clear construct of the social environment. The broad indicators were deleted, as they represented not only social influence, but information sources, delay, intention or provider choice, such as social media trust, waiting for proof, community leader advice, involving girls, recommending vaccination, female-provider preference, and source trust.

Motivation had six indicators (D3_1 to D3_6) that loaded between 0.797 and 0.894. These retained indicators included willingness, willingness to register girls, perceived importance of HPV vaccination, disease-risk motivation, past immunisation experience, and trust in HPV vaccine safety, representing internal readiness for vaccination. D3_7 and D3_8 were eliminated, as they represent external factors rather than factors that drive motivation: scientific information and vaccine use in other countries.

Practical issues retained three indicators (i.e., D4_1, D4_2, and D4_3) with loadings ranging between 0.914 and 0.930. The retained indicators focus on direct contact about HPV vaccination, availability of Pakistani data, and information from healthcare staff. These factors are indicative of the practical readiness and service-related confidence. The deleted indicators include incentives, schools, legal rules, teacher training, outreach, vaccine origin, staff cooperation, access barriers, waiting time, reminders, social media, and media exposure. These mixed subthemes lowered the construct due to not functioning as one narrow reflective dimension.

Cultural integration retained three indicators (i.e., D5_1, D5_3, and D5_4) with loadings ranging between 0.765 and 0.939. The retained indicators focus on household decision-making, educating men, and family opinion. These indicators are suitable for conservative family settings where HPV vaccination decisions include decisions made by family members, awareness of HPV in men, and family approval. The deleted indicators related to women’s health discussion, daughter’s interest, cancer stories, provider gender, stigma, gender focus, cultural background, and parental consent were relevant but represented different cultural and communication dimensions.

The vaccination readiness and uptake composite index retained four indicators (D6_2, D6_3, D6_4, and D6_5), with loadings ranging from 0.831 to 0.905, all statistically significant at *p* < 0.001. The retained indicators capture distinct but related components of the vaccination continuum, including actual HPV vaccination receipt (D6_2), caregiver intention/willingness to vaccinate (D6_3), perceived importance of vaccination (D6_4), and perceived ease of access to vaccination (D6_5). Although the indicators demonstrated strong statistical associations with the composite index, they represent conceptually distinct aspects of vaccination readiness and uptake rather than interchangeable manifestations of a single underlying behavioural construct. Accordingly, the retained indicators were interpreted collectively as a composite index encompassing observed vaccination uptake and proximal dimensions of vaccination readiness, rather than as a measure of actual vaccination uptake alone.

The overall number of deletions of weaker or broader indicators was statistically and practically justified. There were many deleted indicators that were relevant to HPV vaccination but measured different subthemes, including information channels, policy, access, gender norms, stigma, school systems, and media exposure. Their deletion enhanced the clarity, reliability, validity, and parsimony of the constructs. The indicators retained are more specific and coherent measurement model.

Several indicators were deleted in the model refinement process. These items were statistically and practically justified for deletion. While they were relevant to the overarching theme of HPV vaccination, many of the deleted items were conceptually too broad or unrelated to the constructs being explored. These factors were then eliminated, yielding a more accurate final measurement model consisting of statistically and conceptually sound constructs. This process improved the clarity and validity of the latent constructs, making sure that the indicators that were retained were consistent with the theoretical foundation. Thus, the deletion of these items improved the overall reliability, construct validity, and parsimony of the model.

#### 3.6.2. Construct Reliability and Convergent Validity

Table 5 presents the reliability and convergent validity indices for all six latent constructs.

All six constructs demonstrated strong internal consistency and reliability. Cronbach’s alpha coefficients ranged from 0.854 (cultural integration) to 0.928 (thinking and feeling), all comfortably exceeding the 0.80 threshold indicative of good reliability. The Rho_A coefficients ranged from 0.894 to 0.937, and composite reliability values ranged from 0.912 to 0.945—all within the satisfactory range of 0.70–0.95. AVE values ranged from 0.710 (motivation) to 0.850 (practical issues), all exceeding the 0.50 convergent validity threshold, indicating that each construct captured a substantial proportion of variance in its respective indicators.

#### 3.6.3. Goodness-of-Fit

Table 6 presents the model fit indices for the estimated PLS-SEM model.

The SRMR value of 0.079 was at the boundary of the acceptable threshold (<0.08), indicating approximate model fit. Although the NFI (0.684) fell below the threshold of 0.90 traditionally applied in CB-SEM, fit indices in PLS-SEM are considered exploratory and secondary to predictive metrics (R^2^, Q^2^) [13]. The NFI heavily penalises complex structural models with multiple constructs and non-normal data. The model achieved an SRMR of 0.079, meeting the recommended threshold (<0.08) for approximate model fit in PLS-SEM [13]. Combined with high predictive power (R^2^ = 0.658 for motivation, R^2^ = 0.594 for vaccination readiness and uptake composite index) and strong predictive relevance (Q^2^ > 0.35), the measurement and structural model fit is deemed fully acceptable.

The chi-square statistic (6416.95) was large, as expected given the sample size (N = 639) and model complexity, and does not independently indicate poor fit in PLS-SEM. Collectively, these indices suggest an acceptable level of approximate fit for a complex multidimensional model in a challenging empirical context.

### 3.7. Discriminant Validity

#### 3.7.1. Heterotrait–Monotrait (HTMT) Ratio

Table 7 presents the HTMT ratios between all pairs of latent constructs.

All HTMT ratios were below the conservative threshold of 0.90, ranging from 0.492 (TF–VUP) to 0.871 (CINT–PI), confirming that all constructs were empirically distinguishable from one another. The largest HTMT value observed (CINT–PI: 0.871) approached but did not exceed the threshold and was consistent with theoretical expectations, given the conceptual overlap between cultural and practical dimensions of HPV vaccine access.

#### 3.7.2. Fornell–Larcker Criterion (FLC)

Table 8 presents the Fornell–Larcker matrix, with the square root of AVE for each construct on the diagonal.

For each construct, the square root of AVE (bold diagonal) exceeded all corresponding inter-construct correlations, satisfying the Fornell–Larcker criterion across all six constructs. This provides additional confirmation of discriminant validity, indicating that each construct shares more variance with its own indicators than with any other construct in the model.

#### 3.7.3. Cross-Loadings

Table 9 presents the complete cross-loadings matrix for all retained indicators.

For all 27 retained indicators, loadings on their designated constructs exceeded all cross-loadings with other constructs. The patterns of cross-loadings were consistent with the theoretical structure of the model, with indicators demonstrating the expected patterns of discriminant and convergent validity.

#### 3.7.4. Measurement Invariance: MICOM Analysis

The three-step MICOM procedure was conducted to evaluate whether the measurement model is invariant across gender groups (male vs. female) using 1000 permutations. Step 1 (configural invariance) was established by applying identical indicator assignments, model specifications, and algorithm settings across both groups. Step 2 (compositional invariance) confirmed that the composite correlations for five constructs—cultural integration (c = 0.9999, *p* = 0.703), motivation (c = 0.9996, *p* = 0.159), practical issues (c = 0.9999, *p* = 0.396), social process (c = 0.9999, *p* = 0.784), and thinking and feeling (c = 0.9999, *p* = 0.707)—did not differ significantly from 1 (*p* > 0.05), confirming compositional invariance. Vaccination readiness and uptake composite index showed a significant Step 2 *p*-value (*p* = 0.009), indicating configural invariance only for this construct. Step 3 revealed equal composite means and variances for all constructs except motivation (variance *p* = 0.006), establishing full invariance for cultural integration, practical issues, social process, and thinking and feeling, and partial invariance for motivation. Consequently, partial measurement invariance was established across gender groups, validating the use of the model for general cross-gender comparisons and supporting the use of PLS-MGA should group comparisons be required. The MICOM results are reported in detail in Table 10 below.

### 3.8. Structural Model

**Figure 2:**
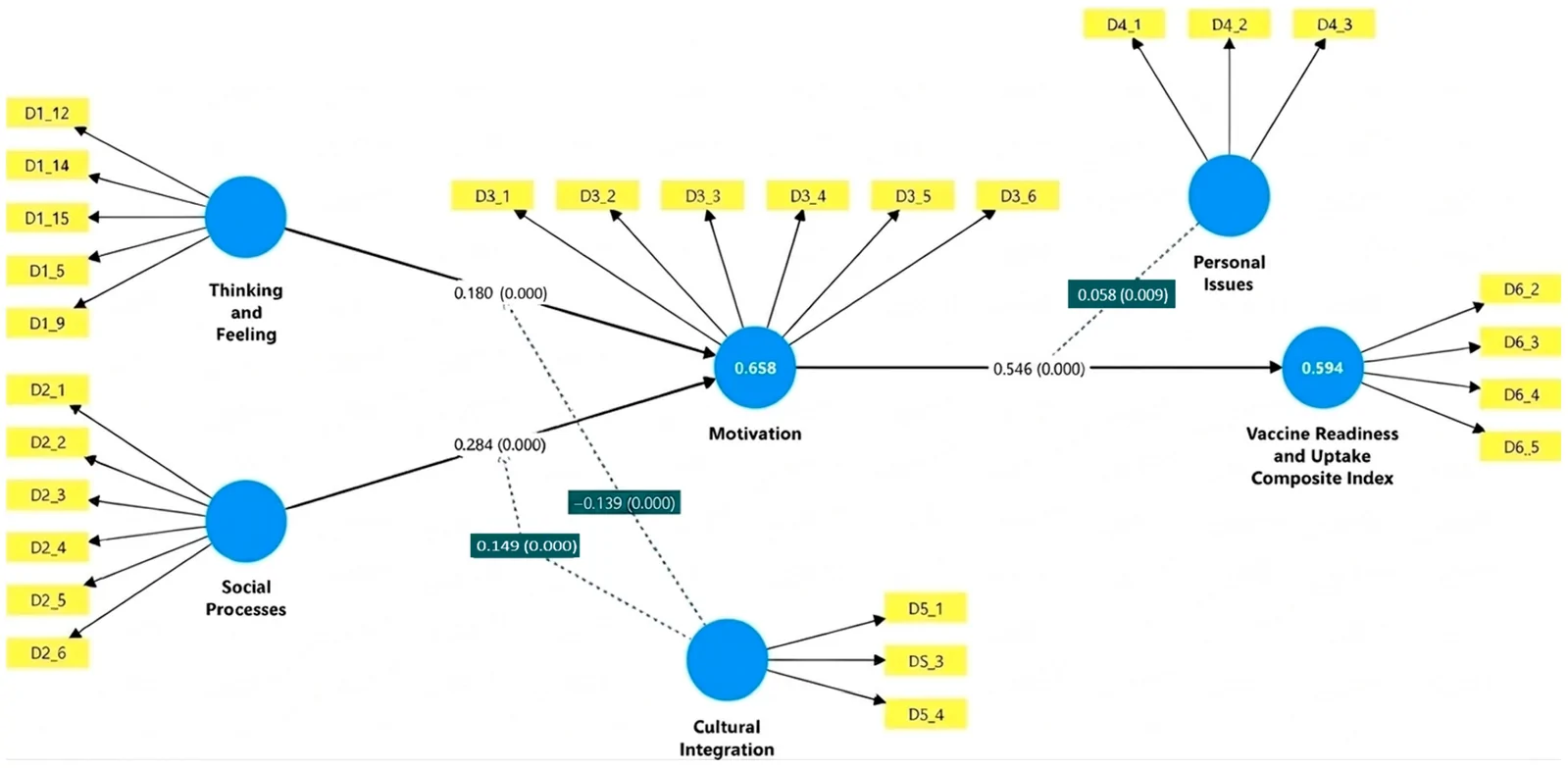
PLS Bootstrapping using SmartPLS. Estimated path coefficients and their corresponding p-values (in parentheses) are shown for direct relationships (solid lines) and moderating interaction effects (dashed lines). Values within the endogenous latent constructs denote the proportion of explained variance (R^2^).

The structural model test aims to assess the postulated relationship between the latent constructs and the explanatory and predictive power of the model [31] (Figure 2).

#### 3.8.1. Direct Effects

Table 11 presents the direct-effect estimates from the PLS-SEM structural model, including the path coefficients, standard deviations, t-statistics, probability values, effect sizes (f^2^), VIF, and 95% BCa confidence intervals.

The thinking and feeling construct is positively and significantly related to motivation (β = 0.180, *p* < 0.05). This means that improvement in thinking and feeling is associated with an increase in motivation toward vaccination uptake. The t-value of 3.955 indicates that this path is significant, and the confidence interval [0.087, 0.265] does not contain zero, reinforcing the significance of this path. The f^2^ value of 0.035 indicates a small effect size.

The social processes construct has a positive and statistically significant relationship with the motivation construct (β = 0.284, *p* < 0.05). This means that better social processes, such as social influence, communication, support, and community interaction, are associated with higher levels of motivation. The t-value is 5.831, which, along with the confidence interval values [0.186, 0.374], indicates that this relationship is strong and significant. The f^2^ value of 0.088 also indicates a small but significant effect size.

Motivation has a positive and statistically significant relationship with the vaccination uptake and composite index constructs (β = 0.546, *p* < 0.05). This finding suggests that the higher the motivation, the higher the rate of vaccination. The t-value of 12.407 suggests a statistically significant effect, and the confidence interval [0.458, 0.629] confirms the positive and statistically significant effect. The f^2^ value of 0.311 reflects a moderate effect size, indicating that motivation has the largest effect in this model.

#### 3.8.2. Indirect Effects (Mediation Analysis)

Table 12 presents the specific indirect effects of motivation. These results show the mediating role of motivation between the independent variables (i.e., social processes and thinking and feeling) and the dependent variable (i.e., vaccination uptake).

The social processes construct showed a significant positive indirect association on vaccination readiness and uptake composite index via motivation (β = 0.155, SD = 0.033, t = 4.745, *p* < 0.001, 95% BCa CI [0.096, 0.222], f^2^ = 0.024). The confidence interval excludes zero, confirming mediation. This finding indicates that the association between social normative processes and vaccination behaviour is substantially accounted for by enhanced motivation.

Similarly, the thinking and feeling construct was indirectly associated with vaccination readiness and uptake composite index via motivation (β = 0.098, SD = 0.024, t = 4.060, *p* < 0.001, 95% CI [0.051, 0.145], f^2^ = 0.010). Both indirect effects were significant, with the social processes pathway exhibiting a comparatively larger effect size.

#### 3.8.3. Moderating Effects

Table 13 presents the results of moderation analysis, investigating the interaction effects of practical issues and cultural integration.

Practical issues positively moderated the motivation → vaccination readiness and uptake composite index relationship (β = 0.058, SD = 0.022, t = 2.621, *p* = 0.009, f^2^ = 0.012, 95% BCa CI [0.015, 0.103]). This indicates that access to service-level information and healthcare staff communication strengthens the association between motivation and vaccination readiness and uptake, though the magnitude of this moderating effect was small.

Cultural integration negatively moderated the thinking and feeling → motivation pathway (β = −0.139, SD = 0.027, t = 5.065, *p* < 0.001, f^2^ = 0.032, 95% BCa CI [−0.189, −0.084]). This suggests that in contexts of stronger cultural integration—where family gatekeeping and patriarchal decision-making norms are pronounced—the association between individual cognitive and affective orientations toward vaccination and personal motivation may be accentuated.

Conversely, cultural integration positively moderated the social processes → motivation pathway (β = 0.149, SD = 0.032, t = 4.707, *p* < 0.001, f^2^ = 0.038, 95% BCa CI [0.084, 0.208]), indicating that cultural embeddedness amplifies the effect of normative social processes on motivation. Collectively, these moderation findings highlight the double-edged role of cultural integration in HPV vaccination decision-making.

#### 3.8.4. Predictive Power and Predictive Relevance

Table 14 presents the R^2^, adjusted R^2^, and Q^2^ values for the endogenous constructs.

The model demonstrated strong predictive power for motivation (R^2^ = 0.658; Adj. R^2^ = 0.655). The model explained 59.4% of the variance in vaccination readiness and uptake (R^2^ = 0.594; Adj. R^2^ = 0.592), representing moderate predictive power. Q^2^ values of 0.650 (motivation) and 0.445 (vaccination readiness and uptake composite index) both exceeded the 0.35 strong relevance threshold, confirming that the model has strong out-of-sample predictive relevance for both endogenous constructs.

#### 3.8.5. PLSpredict Out-of-Sample Predictive Validation

A PLSpredict analysis was conducted using 10-fold cross-validation. The procedure evaluates whether the PLS-SEM model outperforms a naive benchmark (intercept-only average, IA) and a linear regression benchmark (LM) in predicting the endogenous indicator scores on holdout data. The results are presented in Table 15 below. All Q^2^predict values were positive (ranging from 0.327 to 0.506), confirming the model’s predictive relevance beyond the naïve benchmark. However, for all motivation (D3_1 to D3_6), vaccination readiness, and uptake composite index (D6_2 to D6_5) indicators, the PLS-SEM model produced higher RMSE and MAE values than the LM benchmark, indicating that the model lacks out-of-sample predictive power. Therefore, the strength of this model lies primarily in its in-sample explanatory power rather than out-of-sample prediction.

## 4. Discussion

The current study aimed to develop and validate a multidimensional instrument to assess key factors associated with HPV vaccine uptake and readiness in Pakistan using partial least squares structural equation modelling (PLS-SEM). The findings should be interpreted within both the methodological and behavioural context of HPV vaccination research. Methodologically, the work is informed by the principles and recommendations for the appropriate application, evaluation, and interpretation of PLS-SEM described by Hair et al. [32,33]. Conceptually, the study is aligned with the Behavioural and Social Drivers (BeSD) approach to vaccination, which emphasizes the importance of multidimensional behavioural and social determinants in shaping vaccine decision-making. Recent evidence from Zimbabwe further demonstrates the relevance of BeSD domains for understanding HPV vaccination behaviour in low- and middle-income settings [34]. Taken together, these methodological and conceptual foundations provide an appropriate basis for interpreting the present findings and for understanding HPV vaccine uptake and readiness as outcomes influenced by interconnected behavioural, social, and contextual factors. The results indicate that thinking and feeling positively are associated with motivation to get vaccinated. Similar results were portrayed by Chuma et al. who explained that people who have positive thinking about vaccination are motivated. They take time to consider the effectiveness, safety, and benefits of vaccination and hence have greater confidence in their decision. If people think vaccines are beneficial in the prevention of diseases, they are also likely to receive them [34]. Evidence-based evaluation removes uncertainty and boosts readiness and willingness for vaccination. Likewise, Si et al. [35] suggested that this positive mindset increases the odds of behavioural changes in a positive way. When safety replaces fear, people readily accept vaccines. Fernandez et al. [36] supported this result by explaining positive thinking and feeling is positively associated with motivation to get vaccination by balance of rational judgement and emotional comfort. People feel motivated when they know the risk of not getting vaccinated and the benefits of vaccines. The health belief model supports this relationship, as a positive perception and a decrease in concern leads an individual to engage in preventative health activities, including vaccination [37]. However, given the cross-sectional nature of the present study, these findings should be interpreted as associations rather than evidence that thinking and feeling cause greater motivation.

The findings demonstrate a significant positive association between social processes and motivation to vaccinate. These results are supported by Marshall et al. [38], who demonstrated that the social process has a significant effect on motivation to get vaccinated through guidance, reassurance, health information, and interaction with family members, healthcare providers, and communities. In addition, Fernandez et al. [36] reported that supportive social environments were associated with more favourable vaccination decisions by encouraging individuals to get vaccinated. Such an enabling environment is likely to enhance motivation for HPV vaccination by fostering positive perceptions and increasing willingness to be vaccinated, which is consistent with the findings of Si et al. [35], who stated that individual behaviour toward health is largely influenced by social norms and community practices. This phenomenon is supported by social cognitive theory, which provides a theoretical basis for understanding how social norms, observational learning, and social reinforcement may be related to health-related motivation. Nevertheless, the cross-sectional design of this study does not establish that social processes causally increase vaccination motivation.

The findings demonstrate a significant positive association between motivation to vaccinate and the vaccination readiness and uptake. Similar findings were reported by Pot et al. [39], who demonstrated that those people who are internally motivated to get vaccinated are more likely to protect their health through positive steps. People who are motivated, ask questions about vaccination, reduce their hesitation and make vaccination a top priority. Increased readiness to engage in health-related behaviours leads to higher vaccination uptake through increased motivation. Moreover, You et al. [40] likewise reported an association between vaccination motivation and greater consideration of long-term health and safety. In the present study, higher motivation was associated with higher scores on the composite index encompassing vaccination readiness and observed uptake. This finding is broadly consistent with the theory of planned behaviour, in which intention and willingness are theoretically related to subsequent health behaviour. However, because the present study used a cross-sectional design, the observed association should not be interpreted as evidence that motivation causes an increase in vaccination uptake or readiness.

The analysis further indicates a significant indirect association between social processes and the vaccination readiness and uptake through motivation constructs. This finding suggests that the association between social processes and the composite outcome was partly transmitted through differences in reported motivation within the proposed model. This pattern is consistent with previous research indicating that encouragement, social support, and positive vaccination experiences are associated with greater vaccination motivation and vaccine readiness [41]. Si et al. [35] also supported this result and comprehend that motivation is the key energy that drives favourable attitude into positive and impactful action. Those people who feel motivated internally, are more likely to get vaccinated despite hardships like time constrains and concerns. These findings are also theoretically consistent with the theory of planned behaviour, in which social influences are related to behavioural intentions. However, the observed statistical mediation should not be interpreted as confirmation of a causal mechanism or temporal sequence, given the cross-sectional study design.

Similar findings were reported by Krawczyk et al. [41], who pointed out that social processes have a positive impact on people through encouragement, social support, and positive, pleasant experiences about vaccination. Relationships with family, friends, healthcare providers, and communities can be helpful in removing uncertainty and building confidence in vaccines. This positive influence of social circles on individuals thus motivates and builds the desire to actually get vaccinated. Similarly, Si et al. [35] backed this result and stated that the psychological link between social processes and vaccine readiness and uptake is crucial and is mediated by motivation to get vaccinated. Influence from social circles may not influence people directly to vaccinate unless it first increases people willingness and readiness to vaccinate. Marshall et al. [38] also supported this result and found that the mediating role of motivation means that social processes indirectly encourages adoption of vaccination by promoting intention and commitment to vaccination. This relationship is mediated by the theory of planned behaviour, which has shown that social factors impact behavioural intention whereas stronger motivation translates behavioural intention into actual health behaviour, thereby increasing the uptake of vaccination.

The study showed the result that motivation to get vaccinated has positively significantly mediated between thinking and feeling and vaccination uptake. The findings also indicated a significant indirect association between thinking and feeling and the vaccination readiness and Uptake through motivation. This finding suggests that the association between social processes and the composite outcome was partly transmitted through differences in reported motivation within the proposed model. This outcome is in line with Fernandez et al. [36], who stated that both thinking and feeling affect vaccination decisions, impacting on vaccine cognition and vaccine emotions. People are more likely to accept a vaccine if they feel the importance of it, they are reassured and feel secure. Thinking positively decreases the uncertainty and feeling favourably promotes acceptance, which together encourages to enhance motivation to get vaccinated. In addition, similar findings were reported by Si et al. [35] and pointed out that thinking positively and emotional acceptance of vaccines are crucial, and without necessary level of motivation to act, these thinking and feeling do not essentially translate into vaccination. These findings are also theoretically consistent with the theory of planned behaviour, in which social influences are related to behavioural intentions. However, the observed statistical mediation should not be interpreted as confirmation of a causal mechanism or temporal sequence, given the cross-sectional study design. Motivation acts as an internal motivator that converts positive beliefs and feelings into actual intentions and preparedness to accept vaccination and thus boosts vaccination uptake. Pot et al. [39] supported this result and stated that thinking and feeling lead to vaccination uptake indirectly via motivation. Cognitive recognition and emotional feelings of vaccine benefits influence the development of stronger motivation, which is then linked to the generation of more behaviours that support vaccination. This relationship is also explained by the health belief model, which states that when persons have positive intentions about vaccines and their concerns are minor, their motivation to use preventive health measures, including vaccination, is increased. Nevertheless, these findings represent statistical associations within the proposed mediation model and should not be interpreted as evidence that thinking and feeling causally increases vaccination readiness or uptake.

The results showed that practical issues positively moderated the relationship between motivation to get vaccinated and vaccine readiness and uptake, indicating that the positive association between motivation and vaccine readiness and uptake was stronger among individuals experiencing fewer practical barriers.

Similar results were reported by Pot et al. [39], who demonstrated that practical issues have a positive moderating effect on the association between motivation and vaccine readiness and uptake, making it much simpler and easier for motivated individuals to act on their intentions.

When people are motivated internally to get vaccinated, the availability of vaccine clinics, the prices and scheduling of vaccination can make changes between motivation and action. If there is little to no bias against people who are not vaccinated because of their condition, the motivation to be vaccinated has a more significant impact. Similarly, Brewer and Fazekas [21] stated that people might not get vaccinated even if they are motivated due to discrimination because they may face problems regarding long waiting queues, limited facilities, and access to vaccination centres. These hurdles are mitigated in favourable practical conditions, streamlining the achievement of the vaccination process for motivated people. Thus, the motivation to take a vaccine has a stronger effect on its uptake when it is convenient. In addition, You et al. [40] supported this result and concluded that the moderating role of practical issues points to the fact that the effectiveness of motivation in translating into behaviour is influenced by external factors. The health belief model also explains that any kind of health barrier constrain a motivated person to take preventive health measures, hence decreasing vaccine readiness and uptake. However, the moderation finding should be interpreted as an interaction in the observed data rather than evidence that improving practical conditions would causally increase vaccination uptake.

This study offered an operational advantage over the conventional models frequently used in HPV vaccination research. Recent advances in behavioural modelling studies have increasingly emphasised the integration of data-driven analytical techniques with theory-driven approaches, specially in predicting the complex health-related behaviours. Emerging approaches combine statistical explainable analytics with behavioural theory underpinnings to provide strong justification for behavioural and psychosocial determinants that influence vaccine decision-making to improve predictive performance while maintaining model interpretability [42,43]. This study utilised the multidimensional PLS SEM-based approach which has significantly added methodological robustness to identify the predictors of vaccination uptake. Most of the previous studies relied on logistic regression, simple correlation, multiple regression, or logistic regression. Although these models are useful for estimating direct associations, they have limited value in examining complex interrelationships among psychosocial constructs. In contrast, the PLS SEM-based approach simultaneously evaluates structural and measurement models, allowing the measurement of latent constructs with precision while also accounting for measurement errors [13,44]. Moreover, this approach also enables the measurement of direct, mediated, and moderated pathways within a single analytic framework, thereby providing a more realistic representation of factors influencing the vaccination decision-making process.

In the present study, motivation significantly mediated the effects of thinking and feeling and those of social processes on HPV vaccine readiness and uptake, while cultural integration moderated these relationships, demonstrating mechanisms that conventional regression models would not adequately capture. In the present study, motivation mediated the effects of thinking and feeling and those of social processes, while cultural integration moderated these relationships, which are not captured by conventional models adequately.

The current findings need to be understood in the context of prevailing behavioural models and measurement tools grounding HPV vaccination intention. The health belief model (HBM) has been widely utilised with HPV-specific questionnaires that assess perceived susceptibility, perceived severity, perceived benefits and barriers to vaccination, cues to action that encourage preventive behaviour, as well as self-efficacy. Gerend and Shepherd developed and tested measures of HPV vaccination based on the health belief model (HBM) and the theory of planned behaviour (TPB) and in doing so established that perceived susceptibility, severity, benefits, barriers, and physician recommendation, which were included in HBM-based models, were relevant to HPV vaccine readiness and uptake. In addition, attitudes, subjective norms, and self-efficacy derived from TPB assessments were related to intentions to vaccinate against HPV [45].

Likewise, the 14-item health belief model Scale for Human Papillomavirus and its Vaccination (HBMS-HPVV) was developed and psychometrically tested among Turkish nursing students to measure perceived susceptibility, severity, benefits, and barriers [46]. More recently, the Human Papillomavirus Vaccination Scale–health belief model (HPVS-HBM) incorporating constructs like susceptibility, severity, benefits, barriers, and cues to action, has been translated and psychometrically evaluated in Pakistani mothers and has demonstrated good reliability and validity [47].

Measures based on the theory of planned behaviour (TPB) have also been developed, including measures of attitudes, subjective norms, perceived behavioural control and vaccination intentions. For example, studies using TPB with mothers of adolescent children and parents of adolescent girls have shown that attitudes, subjective norms and perceived behavioural control are important in predicting HPV vaccination intentions and behaviour [48]. Purpose-designed TPB questionnaires have also been used in the setting of young women. Subjective norms and perceived behavioural control have been found to be important predictors of intentions to be vaccinated against HPV [49].

Our findings contribute to this evidence base by applying the WHO Behavioural and Social Drivers (BeSD) framework that combines cognitive, social, motivational and practical determinants under one vaccination-specific framework. As opposed to the mostly HBM- or TPB-based instruments that focus on selected psychological constructs or vaccination intentions, the present instrument covers the broader BeSD domains of thinking and feeling, social processes, motivation, and practical issues and empirically investigates their interrelations. Importantly, this study does not only apply the global BeSD survey, but systematically culturally adapts and psychometrically validates the framework for HPV vaccination in Pakistan. Qualitative and validation processes further identified a context-specific domain of cultural integration that included religious beliefs, gender norms, family decision-making, and community acceptance. Rather than developing an entirely new behavioural model, the present study builds upon established HBM- and TPB-derived evidence by culturally adapting and psychometrically validating the WHO BeSD framework and extending its contextual relevance through the identification of a cultural integration domain specific to the Pakistani HPV vaccination context.

The validated multidimensional BeSD-HPV model has wide range of application in epidemiological and implementation research. It can be used to identify behavioural social drivers of vaccination influencing vaccine readiness and uptake, to identify barriers and facilitators influencing vaccine acceptance and to develop targeted evidence-based strategies to promote the vaccination uptake. Another important feature of our instrument was that it extend s beyond traditional HPV vaccination model by incorporating the cultural contextualised construct namely cultural integration, covering patriarchal norms, family decision-making and household approvals. Previous studies on vaccine decision-making mainly focused on knowledge, attitudes, perceived benefits, perceived susceptibility or subjective norms derived from theories such as the theory of planned behaviour and the health belief model, without explicitly incorporating the influence of culturally embedded vaccine decision-making. By incorporating contextualised culturally mediated construct, this framework captures context-specific determinants influencing vaccine readiness and uptake in sociocultural context of conservative society like Pakistan. Thus, the proposed model has not only stronger explanatory power but also greater utility for identifying behavioural and social drivers of vaccination in a culturally mediated context and in resource-limited conditions, with time, cost, and operational constrains. It will guide policy makers and vaccination programme stakeholders to develop targeted social and behavioural change communication (SBCC) interventions and context-specific public health interventions to promote vaccine readiness and uptake among caregivers. In addition, the validated BeSD-HPV model identified significant associations and statistically supported interaction pathways consistent with the proposed theoretical framework, which can be used to evaluate the operational determinants influencing vaccine uptake. However, given the cross-sectional design, the directionality and causality of this association cannot be established. These factors include accessibility, affordability, resource availability, and financial and physical access to vaccination service. Evaluating these factors will help to identify health system barriers that limit vaccine uptake despite a positive attitude and readiness to be vaccinated.

Although partial least squares structural equation modelling (PLS-SEM) is an advanced statistical technique, the proposed model is a feasible option to be implemented in a resource-constrained setting. This methodological expertise is largely confined to a one-time process of construct development and psychometric validation. During the entire process, the researcher evaluated the methodological construct validity, indicator reliability, moderation, mediation, and predictive performance. This methodological robustness was maintained to ensure that the instrument accurately measures behavioural social determinants influencing the HPV vaccination uptake. However, these operational demands are only required during the initial development and psychometric validation phase; once the rigorous validation process has been completed, the resulting 27-item instrument can be used by healthcare workers and vaccination programme stakeholders. Consequently, during routine utilization, the 27-item instrument will be administered to collect data on context-specific behavioural and social determinants that influence vaccine readiness and uptake. Hence, the utilization of validated tools during routine programme use is simple, feasible, cost-effective, and scalable. Therefore, the availability of a reliable, culturally relevant, and psychometrically validated tool that can precisely identify behavioural and social drivers of HPV vaccination offsets the higher initial investment in technical expertise. This allows for more effective intervention targeting and may lower the long-term costs associated with ineffective communication and implementation strategies in low- and middle-income countries.

Recent advances in behavioural modelling have increasingly integrated traditional health behaviour theories with explainable analytics to improve both predictive performance and model interpretability. Rather than relying solely on statistical prediction, contemporary approaches emphasise the transparent identification of the behavioural, social, and contextual factors that drive health-related decisions. Explainable artificial intelligence (XAI) techniques facilitate the interpretation of complex relationships by quantifying the contribution of individual predictors and interactions, thereby supporting more trustworthy and actionable behavioural models. Recent work has highlighted the value of combining theory-driven frameworks with explainable analytical methods to better understand behavioural determinants while maintaining model transparency [50]. These developments support the continued use of theoretically grounded behavioural models alongside interpretable analytical approaches in vaccination research.

### Study Limitations

This study has several limitations that should be considered when interpreting the findings. First, the cross-sectional design precludes establishing temporal or causal relationships among the constructs examined. Although PLS-SEM enables the testing of theoretically specified pathways and indirect effects, the observed associations should not be interpreted as evidence of causality. Longitudinal and prospective studies are needed to confirm the temporal ordering of behavioural and social drivers influencing HPV vaccination uptake.

Second, the participants were recruited using non-probability sampling to ensure representation of diverse socioeconomic, educational, and cultural backgrounds relevant to the early implementation of HPV vaccination in Pakistan. While this approach was appropriate for instrument development and validation, it may limit the representativeness of the sample and introduce selection bias. Consequently, the findings should be generalised cautiously beyond the study population.

Third, all variables were measured using self-reported questionnaires, making the study susceptible to recall bias and social-desirability bias. Participants may have overreported socially acceptable attitudes or vaccination-related behaviours, particularly given the increasing public attention surrounding the national HPV vaccination campaign during the study period.

Fourth, because data for the predictor and outcome constructs were collected using the same instrument at a single point in time, common method bias cannot be completely excluded. Although Harman’s single-factor test indicated that common method variance was unlikely to substantially influence the findings, this statistical approach cannot eliminate the possibility of shared method variance affecting the observed relationships.

Fifth, the substantial refinement of the measurement model resulted in the reduction of the initial 75-item instrument to 27 retained indicators based on psychometric performance and theoretical considerations. While this process improved construct reliability, convergent validity, and model parsimony, it may have reduced the conceptual breadth of some constructs, warranting confirmation in future validation studies using independent samples.

Sixth, the final measurement model was internally validated within a single dataset and was not externally validated using an independent sample. Therefore, the psychometric properties and structural relationships identified in this study should be interpreted as preliminary until replicated in other populations and geographical settings within Pakistan and internationally.

Seventh, although the outcome construct assessed HPV vaccination uptake, several retained indicators also captured behavioural intentions and self-reported vaccination-related behaviours. Because data collection occurred during the first phase of Pakistan’s national HPV vaccination campaign (September–November 2025), not all eligible girls had completed vaccination at the time of assessment. Consequently, the findings should not be interpreted as representing completed vaccination coverage but rather behavioural determinants and early uptake during programme implementation.

Eighth, this study was conducted during a period of rapidly evolving HPV vaccination policy in Pakistan, coinciding with the phased national introduction of the vaccine. Public awareness, service availability, healthcare provider practices, and community perceptions were changing throughout the study period. Therefore, some behavioural determinants identified in this study may evolve as the national programme matures and vaccine availability expands.

Finally, this study was conducted in selected districts of Punjab, including Rawalpindi, Islamabad, and Bahawalpur, chosen to capture sociocultural and geographic diversity during the pre-implementation and early implementation phases of the national HPV vaccination programme. Although these settings provided valuable contextual variation for instrument validation, the findings may not fully represent populations from other provinces, remote rural areas, or regions with different cultural, linguistic, healthcare, and health-system characteristics. Further multicentre validation studies involving nationally representative samples are recommended to establish the broader applicability and measurement invariance of the instrument across diverse Pakistani populations.

An additional limitation relates to the timing of data collection, which coincided with the first phase of Pakistan’s national HPV vaccination campaign (September–November 2025). These campaigns included large-scale public awareness-raising initiatives, community mobilisation, the involvement of healthcare providers, and increased availability of vaccination services across the country. Participants’ attitudes and perceived accessibility of vaccination services, which may influence coverage and self-reported HPV vaccination behaviours, might therefore have been affected not by stable baseline behavioural and social determinants but by the continuous campaign activities. As these contextual factors were not measured separately, their independent effects could not be isolated from the observed associations. For these reasons, the results should be viewed in the context of the initial rollout of the national HPV vaccination programme, and care should be taken when trying to extrapolate them to other time points or interventions in the programme, during which public awareness and service delivery dynamics may have varied.

## 5. Conclusions

This study developed and validated a multidimensional instrument with satisfactory psychometric properties for assessing behavioural and social determinants of HPV vaccination within the Pakistani context. The findings support the instrument’s potential utility for research, programme evaluation, and implementation planning in Pakistan. However, because the instrument was validated using a purposively sampled population from selected locations, further external validation is required before its application can be recommended in other sociocultural settings or low- and middle-income countries. The observed associations may help inform the design of context-specific public health strategies aimed at improving HPV vaccine acceptance and uptake but should not be interpreted as evidence of causal relationships.

This study explicitly presents a validated multidimensional instrument to assess HPV vaccine readiness and uptake determinants in Pakistan using the PLS-SEM analytical framework. The instrument can be used for epidemiological surveys, health behaviour research, and HPV vaccination programmes in resource-limiting settings due to its profound reliability and validity. The research findings indicate that motivation has a central role in vaccination uptake while social processes, thinking and feeling, practical issues, and cultural integration each contribute to shaping this central motivational pathway. Public health information that aims to increase HPV vaccine coverage in Pakistan should prioritise community-level social normative change alongside improvements in healthcare information access, while accounting for the complex moderating influence of cultural integration on individual-level decision-making pathways.

## Figures and Tables

**Table 1:** Pre-testing of the instrument using Cronbach’s alpha (*n* = 50).

Variable	Mean	Std. Dev.	N Items	Cronbach’s Alpha
Cultural Integration	3.432	0.620	12	0.799
Motivation	3.865	0.779	8	0.890
Practical Issues	3.920	0.666	18	0.917
Social Processes	3.437	0.955	13	0.944
Thinking and Feeling	3.052	0.483	19	0.697
Vaccination Readiness and Uptake Composite Index	2.816	0.844	5	0.794

**Table 2:** Demographic profile of the respondents (*n* = 639).

Category	Options	N (%)	Mean ± SD
Gender	Male	157 (24.6%)	
	Female	482 (75.4%)	
Highest education obtained	Primary	68 (10.6%)	
	Secondary	53 (8.3%)	
	Bachelors	157 (24.6%)	
	Intermediate	113 (17.7%)	
	Masters	53 (8.3%)	
	Matric	105 (16.4%)	
	No formal education	88 (13.8%)	
	Others	1 (0.2%)	
	PhD	1 (0.2%)	
Monthly income (in Rupees)	<50,000	195 (30.5%)	
	50,000–100,000	241 (37.7%)	
	>100,000	203 (31.8%)	
Residence	Urban	450(70.4%)	
	Rural	189(29.5%)	
Parent or primary caregiver of any girl (9 to 16 years)	Yes	636 (99.5%)	
	No	3 (0.5%)	
Your relationship with the girl	Mother	433 (67.8%)	
	Father	193 (30.2%)	
	Grand mother	1 (0.15%)	
	Grand father	1 (0.15%)	
	Aunt	6 (0.93%)	
	Uncle	5 (0.72%)	
Has your girl received first dose of HPV vaccine?	Yes	400 (62.5%)	
	No	201 (31.5%)	
	Don’t Know	38 (5.9%)	
Age (in years)			54.19 ± 17.714
Total no. of children			3.54 ± 1.375
How many girls do you have who are between 9 to 16 years of age?			1.65 ± 0.956
Age of girl (in years)			12.60 ± 3.440

**Table 3:** Descriptive statistics for domains D1 to D6 of the study constructs (N = 639).

Item	Mean	SD	Skewness	Kurtosis	KS (Prob.)	SW (Prob.)
**Thinking and Feeling (D1; 19 items)**						
**D1_1**	2.86	1.235	0.055	−0.835	0.186 (<0.001)	0.906 (<0.001)
**D1_2**	2.95	1.124	0.112	−0.505	0.212 (<0.001)	0.908 (<0.001)
**D1_3**	3.19	1.158	−0.197	−0.684	0.173 (<0.001)	0.913 (<0.001)
**D1_4**	3.10	1.141	−0.181	−0.578	0.200 (<0.001)	0.910 (<0.001)
**D1_5**	2.95	1.130	−0.003	−0.550	0.206 (<0.001)	0.910 (<0.001)
**D1_6**	2.84	1.043	0.067	−0.463	0.198 (<0.001)	0.912 (<0.001)
**D1_7**	3.04	1.117	−0.111	−0.536	0.205 (<0.001)	0.911 (<0.001)
**D1_8**	2.93	1.090	−0.035	−0.634	0.186 (<0.001)	0.915 (<0.001)
**D1_9**	3.00	1.097	−0.229	−0.551	0.215 (<0.001)	0.905 (<0.001)
**D1_10**	2.89	1.152	0.092	−0.691	0.176 (<0.001)	0.915 (<0.001)
**D1_11**	3.05	1.203	−0.154	−0.821	0.175 (<0.001)	0.912 (<0.001)
**D1_12**	2.92	1.157	−0.121	−0.582	0.237 (<0.001)	0.895 (<0.001)
**D1_13**	2.92	1.176	−0.138	−0.787	0.202 (<0.001)	0.906 (<0.001)
**D1_14**	3.06	1.152	−0.231	−0.559	0.221 (<0.001)	0.901 (<0.001)
**D1_15**	2.79	1.172	0.126	−0.577	0.213 (<0.001)	0.897 (<0.001)
**D1_16**	3.90	0.698	−0.283	0.022	0.306 (<0.001)	0.820 (<0.001)
**D1_17**	3.87	0.698	−0.599	0.729	0.345 (<0.001)	0.793 (<0.001)
**D1_18**	3.97	0.735	−0.553	0.397	0.309 (<0.001)	0.815 (<0.001)
**D1_19**	3.51	0.956	−0.318	−0.555	0.252 (<0.001)	0.886 (<0.001)
**Social Processes (D2; 13 items)**						
**D2_1**	3.17	1.043	−0.454	−0.147	0.233 (<0.001)	0.886 (<0.001)
**D2_2**	3.26	1.018	−0.457	−0.225	0.217 (<0.001)	0.892 (<0.001)
**D2_3**	3.20	1.031	−0.314	−0.183	0.220 (<0.001)	0.899 (<0.001)
**D2_4**	3.22	1.028	−0.150	−0.431	0.189 (<0.001)	0.910 (<0.001)
**D2_5**	3.09	0.968	−0.085	0.077	0.249 (<0.001)	0.888 (<0.001)
**D2_6**	3.28	0.989	−0.045	−0.374	0.220 (<0.001)	0.904 (<0.001)
**D2_7**	3.61	0.973	−0.954	0.787	0.308 (<0.001)	0.833 (<0.001)
**D2_8**	3.05	1.148	−0.525	−1.034	0.292 (<0.001)	0.828 (<0.001)
**D2_9**	3.63	0.886	−0.749	0.411	0.316 (<0.001)	0.841 (<0.001)
**D2_10**	3.42	1.049	−0.640	−0.209	0.277 (<0.001)	0.871 (<0.001)
**D2_11**	3.75	0.884	−0.383	−0.514	0.269 (<0.001)	0.863 (<0.001)
**D2_12**	4.11	0.638	−0.314	0.267	0.314 (<0.001)	0.782 (<0.001)
**D2_13**	3.98	0.631	−0.589	1.381	0.353 (<0.001)	0.754 (<0.001)
**Motivation (D3; 8 items)**						
**D3_1**	3.30	1.016	−0.511	−0.078	0.218 (<0.001)	0.888 (<0.001)
**D3_2**	3.31	0.973	−0.393	0.138	0.222 (<0.001)	0.886 (<0.001)
**D3_3**	3.19	0.938	−0.438	−0.193	0.217 (<0.001)	0.883 (<0.001)
**D3_4**	3.17	0.984	−0.373	0.224	0.263 (<0.001)	0.873 (<0.001)
**D3_5**	3.16	0.937	−0.116	0.145	0.248 (<0.001)	0.886 (<0.001)
**D3_6**	2.98	0.965	−0.210	−0.126	0.247 (<0.001)	0.894 (<0.001)
**D3_7**	3.92	0.698	−0.667	0.936	0.347 (<0.001)	0.784 (<0.001)
**D3_8**	3.95	0.671	−0.499	0.728	0.335 (<0.001)	0.790 (<0.001)
**Practical Issues (D4; 18 items)**						
D4_1	3.08	1.187	−0.061	−0.762	0.173 (<0.001)	0.914 (<0.001)
D4_2	3.13	1.268	−0.296	−0.891	0.181 (<0.001)	0.897 (<0.001)
D4_3	2.95	1.074	0.016	−0.509	0.195 (<0.001)	0.914 (<0.001)
D4_4	3.01	1.187	−0.032	−0.824	0.161 (<0.001)	0.916 (<0.001)
D4_5	3.08	1.048	−0.162	−0.425	0.205 (<0.001)	0.910 (<0.001)
D4_6	2.74	1.314	0.134	−1.231	0.191 (<0.001)	0.889 (<0.001)
D4_7	4.07	0.819	−0.584	−0.234	0.245 (<0.001)	0.832 (<0.001)
D4_8	3.28	1.118	−0.191	−0.835	0.207 (<0.001)	0.907 (<0.001)
D4_9	3.22	1.184	−0.246	−0.942	0.224 (<0.001)	0.901 (<0.001)
D4_10	3.70	1.091	−0.697	−0.268	0.271 (<0.001)	0.864 (<0.001)
D4_11	4.23	0.687	−0.449	−0.381	0.260 (<0.001)	0.795 (<0.001)
D4_12	4.29	0.620	−0.288	−0.644	0.301 (<0.001)	0.764 (<0.001)
D4_13	4.33	0.609	−0.331	−0.656	0.300 (<0.001)	0.753 (<0.001)
D4_14	4.24	0.664	−0.312	−0.778	0.272 (<0.001)	0.784 (<0.001)
D4_15	4.36	0.652	−0.521	−0.688	0.293 (<0.001)	0.761 (<0.001)
D4_16	3.98	0.638	−0.202	0.125	0.318 (<0.001)	0.790 (<0.001)
D4_17	4.11	0.574	<0.001	−0.063	0.350 (<0.001)	0.745 (<0.001)
D4_18	4.11	0.551	0.052	0.166	0.366 (<0.001)	0.726 (<0.001)
**Cultural Integration (D5; 12 items)**						
D5_1	3.50	1.096	−0.417	−0.416	0.200 (<0.001)	0.899 (<0.001)
D5_2	3.33	1.031	−0.171	−0.543	0.188 (<0.001)	0.908 (<0.001)
D5_3	3.28	1.134	−0.310	−0.655	0.202 (<0.001)	0.908 (<0.001)
D5_4	3.18	1.123	−0.079	−0.637	0.187 (<0.001)	0.914 (<0.001)
D5_5	3.89	0.714	−0.305	−0.003	0.302 (<0.001)	0.826 (<0.001)
D5_6	3.94	0.724	−0.574	0.541	0.324 (<0.001)	0.807 (<0.001)
D5_7	3.85	0.706	−0.534	0.523	0.337 (<0.001)	0.804 (<0.001)
D5_8	3.84	0.803	−0.422	−0.164	0.288 (<0.001)	0.847 (<0.001)
D5_9	3.85	0.777	−0.944	1.944	0.330 (<0.001)	0.802 (<0.001)
D5_10	3.98	0.719	−0.658	0.786	0.327 (<0.001)	0.795 (<0.001)
D5_11	4.00	0.720	−0.957	1.604	0.360 (<0.001)	0.743 (<0.001)
D5_12	3.96	0.618	−0.692	1.760	0.371 (<0.001)	0.735 (<0.001)
**Vaccination Readiness and Uptake Composite Index (D6; 5 items)**						
D6_1	3.48	0.863	−0.081	0.161	0.257 (<0.001)	0.865 (<0.001)
D6_2	3.24	0.891	−0.105	0.091	0.245 (<0.001)	0.885 (<0.001)
D6_3	3.23	0.994	−0.144	−0.288	0.206 (<0.001)	0.905 (<0.001)
D6_4	3.33	1.001	−0.201	−0.170	0.215 (<0.001)	0.899 (<0.001)
D6_5	3.32	0.927	0.067	−0.301	0.247 (<0.001)	0.891 (<0.001)

Note. KS = Kolmogorov–Smirnov test statistic; SW = Shapiro–Wilk test statistic; values in parentheses denote *p*-values. All KS and SW tests reached statistical significance (*p* < 0.001), indicating departure from normality at the item level. SD = standard deviation. Items are grouped by construct domain.

**Table 4:** Indicator reliability and multicollinearity assessment (PLS algorithm).

Latent Construct/Indicator	Loading	Prob.	VIF	Status
**Thinking and Feeling**				
**D1_5**	0.861	<0.001	2.863	Retained
**D1_9**	0.877	<0.001	3.344	Retained
**D1_12**	0.878	<0.001	2.849	Retained
**D1_14**	0.922	<0.001	4.180	Retained
**D1_15**	0.866	<0.001	2.658	Retained
**Social Processes**				
**D2_1**	0.793	<0.001	2.323	Retained
**D2_2**	0.855	<0.001	3.065	Retained
**D2_3**	0.870	<0.001	3.697	Retained
**D2_4**	0.863	<0.001	3.111	Retained
**D2_5**	0.895	<0.001	3.704	Retained
**D2_6**	0.813	<0.001	2.362	Retained
**Motivation**				
**D3_1**	0.834	<0.001	3.989	Retained
**D3_2**	0.835	<0.001	4.479	Retained
**D3_3**	0.797	<0.001	2.821	Retained
**D3_4**	0.850	<0.001	3.245	Retained
**D3_5**	0.894	<0.001	4.236	Retained
**D3_6**	0.842	<0.001	3.265	Retained
**Practical Issues**				
**D4_1**	0.921	<0.001	3.187	Retained
**D4_2**	0.930	<0.001	3.315	Retained
**D4_3**	0.914	<0.001	2.847	Retained
**Cultural Integration**				
**D5_1**	0.765	<0.001	1.578	Retained
**D5_3**	0.928	<0.001	3.470	Retained
**D5_4**	0.939	<0.001	3.867	Retained
**Vaccination Readiness and Uptake Composite Index**				
**D6_2**	0.831	<0.001	1.981	Retained
**D6_3**	0.905	<0.001	3.290	Retained
**D6_4**	0.899	<0.001	3.570	Retained
**D6_5**	0.874	<0.001	2.762	Retained

Note. All retained indicators are statistically significant at *p* < 0.001. VIF = variance inflation factor. Values in bold represent the final retained indicator set. Loading threshold ≥ 0.70 [13]. VIF threshold < 5.0.

**Table 5:** Construct reliability and convergent validity indices.

Latent Construct	Cronbach’s α	Rho_A	Composite Reliability	AVE
**Cultural Integration**	0.854	0.894	0.912	0.776
**Motivation**	0.918	0.922	0.936	0.710
**Practical Issues**	0.911	0.913	0.944	0.850
**Social Processes**	0.922	0.928	0.939	0.721
**Thinking and Feeling**	0.928	0.937	0.945	0.776
**Vaccination Readiness and Uptake Composite Index**	0.900	0.901	0.931	0.771

Note. α = Cronbach’s alpha; Rho_A = Dijkstra–Henseler’s rho; AVE = average variance extracted. Recommended thresholds: α ≥ 0.70, Rho_A ≥ 0.70, CR: 0.70–0.95, AVE ≥ 0.50 [13].

**Table 6:** Model fit indices.

Fit Index	Estimated Model Value
**Standardised Root Mean Square Residual (SRMR)**	0.079
**Squared Euclidean Distance (d_ULS)**	2.343
**Geodesic Distance (d_G)**	2.203
**Chi-Square (χ^2^)**	6416.950
**Normed Fit Index (NFI)**	0.684

Note. SRMR = standardised root mean square residual (acceptable < 0.08); NFI = normed fit index (desirable ≥ 0.90); d_ULS = squared Euclidean distance; d_G = geodesic distance; χ^2^ = chi-square (sensitive to sample size). Fit assessment follows [13].

**Table 7:** Discriminant validity: the Heterotrait–Monotrait (HTMT) ratio.

	CINT	MOT	PI	SP	TF	VUP
**CINT**	**—**					
**MOT**	0.839	**—**				
**PI**	0.871	0.825	**—**			
**SP**	0.782	0.755	0.713	**—**		
**TF**	0.753	0.711	0.562	0.803	**—**	
**VRUCI**	0.642	0.813	0.751	0.597	0.492	**—**

Note. CINT = cultural integration; MOT = motivation; PI = practical issues; SP = social processes; TF = thinking and feeling; VRUCI = vaccination readiness and uptake composite index values below the diagonal. HTMT threshold < 0.90 [29]. Dashes (—) indicate self-referential cells.

**Table 8:** Discriminant validity: the Fornell–Larcker criterion.

	CINT	MOT	PI	SP	TF	VUR
**CINT**	**0.881**					
**MOT**	0.754	**0.843**				
**PI**	0.767	0.755	**0.922**			
**SP**	0.683	0.703	0.658	**0.849**		
**TF**	0.676	0.672	0.524	0.751	**0.881**	
**VRUCI**	0.561	0.745	0.683	0.550	0.457	**0.878**

Note. Bold underlined diagonal values represent the square root of AVE for each construct. Off-diagonal values are inter-construct correlations. Discriminant validity is supported when diagonal values exceed all corresponding off-diagonal values in the same row and column [30]. CINT = cultural integration; MOT = motivation; PI = practical issues; SP = social processes; TF = thinking and feeling; VRUCI = vaccination readiness and uptake composite index.

**Table 9:** Discriminant validity: the cross-loadings matrix.

Indicator	TF	SP	MOT	PI	CINT	VRUCI
**D1_12**	**0.878**	0.718	0.674	0.528	0.593	0.439
**D1_14**	**0.922**	0.735	0.627	0.535	0.683	0.417
**D1_15**	**0.866**	0.651	0.617	0.436	0.583	0.449
**D1_5**	**0.861**	0.588	0.538	0.406	0.580	0.384
**D1_9**	**0.877**	0.580	0.458	0.367	0.516	0.293
**D2_1**	0.599	**0.793**	0.464	0.433	0.473	0.391
**D2_2**	0.698	**0.855**	0.576	0.542	0.621	0.469
**D2_3**	0.692	**0.870**	0.638	0.587	0.716	0.458
**D2_4**	0.610	**0.863**	0.663	0.633	0.538	0.603
**D2_5**	0.696	**0.895**	0.621	0.499	0.589	0.465
**D2_6**	0.528	**0.813**	0.586	0.630	0.525	0.390
**D3_1**	0.497	0.550	**0.834**	0.587	0.650	0.608
**D3_2**	0.464	0.554	**0.835**	0.665	0.657	0.556
**D3_3**	0.495	0.579	**0.797**	0.648	0.599	0.520
**D3_4**	0.629	0.594	**0.850**	0.608	0.584	0.685
**D3_5**	0.606	0.686	**0.894**	0.703	0.671	0.725
**D3_6**	0.683	0.580	**0.842**	0.610	0.650	0.651
**D4_1**	0.529	0.625	0.647	**0.921**	0.766	0.604
**D4_2**	0.463	0.578	0.715	**0.930**	0.656	0.650
**D4_3**	0.461	0.618	0.724	**0.914**	0.704	0.634
**D5_1**	0.550	0.681	0.509	0.603	**0.765**	0.477
**D5_3**	0.650	0.606	0.767	0.702	**0.928**	0.562
**D5_4**	0.584	0.554	0.681	0.721	**0.939**	0.445
**D6_2**	0.386	0.487	0.675	0.656	0.482	**0.831**
**D6_3**	0.392	0.539	0.645	0.606	0.465	**0.905**
**D6_4**	0.435	0.475	0.677	0.541	0.501	**0.899**
**D6_5**	0.391	0.424	0.614	0.589	0.523	**0.874**

Note. Bold values represent the highest loading for each indicator (i.e., loading on its designated construct). Discriminant validity is supported when each indicator’s loading on its own construct exceeds all cross-loadings [13]. TF = thinking and feeling; SP = social processes; MOT = motivation; PI = practical issues; CINT = cultural integration; VRUCI = vaccination readiness and uptake composite index.

**Table 10:** Measurement invariance assessment (MICOM) results across gender groups (male vs. female).

Construct	Step 1: Config.	Step 2: Comp. Corr. (c)	5% Quantile	Step 2 *p*-Value	Step 2 Established?	Step 3a: Mean Diff.	Step 3a *p*-Value	Step 3b: Var. Diff.	Step 3b *p*-Value	Overall Invariance
Cultural Integration	Yes	0.9999	0.9985	0.703	Yes	−0.016	0.859	−0.136	0.284	Full Invariance
Motivation	Yes	0.9996	0.9994	0.159	Yes	0.012	0.891	−0.387	0.006	Partial Invariance
Practical Issues	Yes	0.9999	0.9997	0.396	Yes	−0.085	0.353	0.086	0.374	Full Invariance
Social Process	Yes	0.9999	0.9991	0.784	Yes	0.068	0.457	−0.088	0.492	Full Invariance
Thinking and Feeling	Yes	0.9999	0.9995	0.707	Yes	0.098	0.289	−0.130	0.251	Full Invariance
Vaccine Readiness and Uptake Composite Index	Yes	0.9994	0.9997	0.009	No	−0.039	0.697	−0.098	0.509	Configural Only

Note: c = composite correlation across data groups. Permutation test conducted with 1000 permutations. Step 1 (configural invariance) is established qualitatively by design. Step 2 (compositional invariance) is established when the original correlation c is ≥ the 5% quantile threshold, and the permutation *p*-value is >0.05. Step 3a (equal means) and Step 3b (equal variances) are established when permutation *p*-values are >0.05. Full measurement invariance requires Steps 1, 2, 3a, and 3b. Partial invariance requires Steps 1 and 2.

**Table 11:** Direct-effect analysis.

Path	β	SD	t-Stats	*p*-Value	f^2^	VIF	2.5–97.5% CI
**TF → MOT**	0.180	0.046	3.955	<0.001	0.035	2.749	[0.087–0.265]
**SP → MOT**	0.284	0.049	5.831	<0.001	0.088	2.676	[0.186–0.374]
**MOT → VRUCI**	0.546	0.044	12.407	<0.001	0.311	2.358	[0.458–0.629]

Note. β = standardised path coefficient; SD = standard deviation; f^2^ = Cohen’s effect size (small: 0.02; medium: 0.15; large: 0.35); VIF = variance inflation factor; CI = bias-corrected and accelerated confidence interval. TF = thinking and feeling; SP = social processes; MOT = motivation; VRUCI = vaccination readiness and uptake composite index. All VIF values < 5.0 indicate no multicollinearity concern.

**Table 12:** Specific indirect-effect analysis (mediation via motivation).

Indirect Path	β	SD	t-Stats	*p*-Value	f^2^	2.5–95%CI
**SP → MOT → VRUCI**	0.155	0.033	4.745	<0.001	0.024	[0.096 5%CIthir
**TF → MOT → VRUCI**	0.098	0.024	4.060	<0.001	0.010	[0.051 5%CIthir

Note. β = standardised indirect path coefficient; SD = standard deviation; f^2^ = Cohen’s effect size; CI = bias-corrected and accelerated confidence interval. SP = social processes; TF = thinking and feeling; MOT = motivation; VURCI = vaccination readiness and uptake composite index. Mediation is supported when confidence intervals exclude zero.

**Table 13:** Moderating-effect analysis (PLS-SEM).

Interaction Term	β	SD	t-Stat	*p*-Value	f^2^	VIF	95% CI
**PI × MOT → VRUCI**	0.058	0.022	2.621	0.009	0.012	1.015	[0.015–0.103]
**CINT × TF → MOT**	−0.139	0.027	5.065	<0.001	0.032	3.012	[−0.189–−0.084]
**CINT × SP → MOT**	0.149	0.032	4.707	<0.001	0.038	2.938	[0.084–0.208]

Note. β = standardised interaction path coefficient; SD = standard deviation; f^2^ = Cohen’s effect size; VIF = variance inflation factor; CI = bias-corrected and accelerated confidence interval. PI = practical issues; CINT = cultural integration; MOT = motivation; TF = thinking and feeling; SP = social processes; VRUCI = vaccination readiness and uptake composite index. A negative β for CINT × TF → MOT indicates the dampening of the TF–MOT relationship at higher levels of cultural integration.

**Table 14:** Predictive power (R^2^) and predictive relevance (Q^2^) of endogenous constructs.

Endogenous Construct	R^2^	Adj. R^2^	Q^2^ (Predictive Relevance)	Predictive Power
**Motivation**	0.658	0.655	0.650	Strong relevance
**Vaccination Readiness and Uptake Composite Index**	0.594	0.592	0.445	Strong relevance

Note. R^2^ = coefficient of determination; Adj. R^2^ = adjusted R^2^; Q^2^ = Stone–Geisser’s predictive relevance (blindfolding procedure). Thresholds for R^2^: weak ≈ 0.25, moderate ≈ 0.50, substantial ≈ 0.75 [17]. Thresholds for Q^2^: weak = 0.02, moderate = 0.15, strong = 0.35 [18].

**Table 15:** PLSpredict out-of-sample predictive performance (10-fold cross-validation).

Indicator	Q^2^predict	PLS-SEM RMSE	PLS-SEM MAE	LM RMSE	LM MAE	IA RMSE	IA MAE
D3_1	0.434	0.765	0.584	0.591	0.471	1.017	0.828
D3_2	0.448	0.724	0.553	0.627	0.528	0.974	0.780
D3_3	0.426	0.711	0.530	0.590	0.481	0.939	0.757
D3_4	0.442	0.735	0.547	0.615	0.489	0.985	0.727
D3_5	0.506	0.659	0.507	0.480	0.372	0.938	0.698
D3_6	0.506	0.679	0.538	0.428	0.322	0.966	0.680
D6_2	0.361	0.713	0.564	0.486	0.376	0.892	0.703
D6_3	0.345	0.805	0.606	0.647	0.520	0.995	0.797
D6_4	0.327	0.822	0.646	0.674	0.547	1.002	0.814
D6_5	0.328	0.760	0.609	0.628	0.480	0.928	0.761

Note. Q^2^predict = Stone–Geisser’s predictive relevance index; positive values indicate the PLS-SEM model outperforms the naïve intercept-only average (IA). PLS-SEM RMSE/MAE = prediction errors of the PLS-SEM model on holdout data; LM RMSE/MAE = prediction errors of the linear model benchmark; IA = intercept-only average baseline. D3 = motivation indicators; D6 = vaccination readiness and uptake composite index indicators.

## Data Availability

Researchers seeking access to the underlying raw data may contact the corresponding author to request it.

## Supplementary Materials

The following supporting information can be downloaded at: https://www.mdpi.com/article/10.3390/vaccines14090761/s1, File S1: Final Questionnaire with urdu translation (70 Items) as in Kobo toolbox. File S2: Item Reduction and Content Validity Table. File S3: Modified Survey after Analysis (27 Item).
