# Supplementary material for: Development and Initial Validation of a Multidimensional Instrument for Assessing Determinants of HPV Vaccine Readiness and Uptake Among Caregivers in Pakistan: A PLS-SEM Approach
Source: Vaccines (Basel). 2026 Aug 31;14(9):761. doi: 10.3390/vaccines14090761 (PMC13611574; doi:10.3390/vaccines14090761)
Supplement: Supplementary file 1 [file vaccines-14-00761-s001.zip › vaccines-4419243-supplementary/Supplementary File S1.pdf]

Final Questionnaire with urdu translation (70 Items) as in Kobo toolbox

	**Domain 1: Thinking and Feeling**
1	**How safe do you think HPV vaccine is for your daughter?**<br>آپ کے خیال میں ایچ پی وی ویکسین آپ کی بیٹی کے لیے کتنی محفوظ ہے؟
2	**How much do you trust the health workers who give HPV vaccines?**<br>آپ ایچ پی وی ویکسین دینے والے صحت کے کارکنان پر کتنا اعتماد کرتے ہیں؟
3	To what extent do you agree that preventive measures, such as vaccination, are necessary to protect women from serious illnesses?<br>آپ اس بات سے کس حد تک متفق ہیں کہ خواتین میں سنگین بیماریوں سے بچاؤ کے لیے حفاظتی اقدامات، جیسے کہ ویکسینیشن، ضروری ہیں؟
4	In your opinion, could the vaccines being administered in Pakistan be used for experimental purposes?<br>کیا آپ اس بات سے متفق ہیں کہ پاکستان میں دی جانے والی ویکسین کو تجرباتی مقاصد کے لیے استعمال کیا جا سکتا ہے ؟
5	In your opinion, do vaccines contain ingredients that are prohibited in religion (pork or alcohol)?<br>کیا آپ کے خیال میں ویکسینز میں ایسے اجزاء موجود ہیں جو مذہبی اعتبار سے ممنوع ہیں؟
6	To what extent do you think that the HPV vaccine beneficial to the women in Pakistan?<br>آپ کے خیال میں پاکستان میں خواتین کے لئے ایچ پی وی ویکسین کس حد تک فائدہ مند ہے ؟
7	To what extent do you believe that the HPV vaccine protects against cervical cancer, based on your existing knowledge?<br>آپ کی موجودہ معلومات کے مطابق آپ کس حد تک سمجھتے ہیں کہ ایچ پی وی ویکسین سروائیکل کینسر کے خلاف تحفظ فراہم کرتی ہے؟
8	Do you believe is your current knowledge sufficient to inform you regarding vaccinating against HPV?<br>آپ کس حد تک سمجھتے ہیں کہ آپ کی موجودہ معلومات ایچ پی وی ویکسین لگوانے کا فیصلہ کرنے کے لیے کافی ہے ؟
9	How much do you trust the HPV vaccine being introduced in your country?<br>آپ اپنے ملک میں متعارف کی جانے والی ایچ پی وی ویکسین پر کتنا اعتماد کرتے ہیں؟
10	Do you think that if the government makes the HPV vaccine mandatory, some people will try to get a card without actually being vaccinated due to their biases?<br>کیا آپ سمجھتے ہیں کہ اگر حکومت ایچ پی وی ویکسین لازمی قرار دے دے تو کچھ لوگ اپنے تعصبات کی وجہ سے ویکسین لگوائے بغیر ہی کارڈ حاصل کرنے کی کوشش کریں گے ؟
11	If the HPV vaccine were offered free of cost, do you think people would receive it?<br>کیا آپ سمجھتے ہیں کہ اگر ایچ پی وی ویکسین مفت دی جائے تو لوگ اسے لگوانے میں ہچکچاہٹ محسوس کریں گے ؟
12	Do you believe the HPV vaccine is acceptable in a conservative society?<br>کیا آپ سمجھتے ہیں کہ ایچ پی وی ویکسین ایک قدیم نظریات رکھنے والے معاشرے میں قابل قبول ہے ؟
13	To what extent do you agree that all girls aged 9 to 14 years should be vaccinated against HPV?<br>آپ کس حد تک ایچ پی وی اس بات سے متفق ہیں کہ ۹ سے ۱۴ سال کی تمام لڑکیوں کو ویکسین لگوانی چاہئے؟
14	If you think the women in your family are at low risk of cervical cancer due to family history, would you still vaccinate them against HPV?

	اپنے خاندانی پس منظر کو پیش نظر رکھتے ہوئے اگر آپ کے خاندان کی عورتوں کو سرویکل کینسر کا خطرہ کم ہو تو کیا آپ پھر بھی ان کو ایچ پی وی ویکسین لگوائیں گے ؟
15	Do you think the HPV vaccine will help protect women in your family from serious illness?<br>کیا آپ سمجھتے ہیں کہ ایچ پی وی ویکسین آپ کے خاندان کی عورتوں کوسنگین بیماری سے بچانے میں مددگار ثابت ہو گی ؟
16	To what extent do you think a person's strong natural immunity affects their decision to receive the HPV vaccine?<br>کیا آپ سمجھتے ہیں کہ مضبوط قوت مدافعت ایچ پی وی ویکسین لگوانے کے فیصلے پر اثر انداز ہو گی ؟
17	To what extent are you concerned about the possible adverse effects of the HPV vaccine?<br>آپ کس حد تک ایچ پی وی ویکسین کے ممکنہ موذی اثرات کے بارے میں فکر مند ہیں؟
18	To what extent do you think the HPV vaccine could negatively affect reproductive health?<br>کیا آپ سمجھتے ہیں کہ ایچ پی وی ویکسین تولیدی صحت پر منفی اثارت ڈال سکتی ہے
19	How much do you trust social media to get information about the HPV vaccine?<br>یچ پی وی ویکسین کے بارے میں معلومات حاصل کرنے کے لیے آپ سوشل میڈیا پر کتنا بھروسہ کرتے ہیں ؟
	**Domain 2: Social Processes**
**20**	**Do you think most parents you know would want you to get your daughter vaccinated against HPV ?**<br>آپ کیا سمجھتے ہیں کہ زیادہ تر والدین جنہیں آپ جانتے ہیں وہ یہ چاہیں گے کہ آپ اپنی بیٹی کو ایچ پی وی ویکسین لگوائیں
**21**	**Do you think most of your close family and friends want you to get your daughter vaccinated against HPV?**<br>آپ کیا سمجھتے ہیں کہ آپ کے نزدیکی گھر والے اور دوست یہ چاہیں گے کہ آپ اپنی بیٹی کو ایچ پی وی ویکسین لگوائیں
**22**	**Has a health worker recommended your daughter vaccinated against HPV?**<br>کیا صحت سے تعلق رکھنے والے کارکنوں نے آپ کی بیٹی کو ایچ پی وی ویکسین تجویز کی ہے
**23**	**If it was time for your daughter to get vaccinated, would the mother need permission to take her to the clinic?**<br>اگر آپ کی بیٹی کو ویکسین لگنے کا وقت ہو تو کیا اس کی والدہ کو کسی کی اجازت کی ضرورت ہو گی
24	How often do you hear rumors or misconceptions about the HPV vaccine in your community?<br>آپکو اپنی کمیونٹی میں ایچ پی وی ویکسین سے متعلق افواہیں یا غلط فہمیاں کتنی بار سُننے کو ملتی ہیں؟
25	To what extent do religious beliefs in your community influence decisions about the HPV vaccine?<br>آپ کی کمیونٹی میں مذہبی عقائد کس حد تک ایچ پی وی ویکسین سے متعلق فیصلوں پر اثر انداز ہوتے ہیں؟
26	.To what extent do people in your community trust information about the HPV vaccine shared on social media platforms?<br>کیا آپ کی کمیونٹی کے لوگ سوشل میڈیا پر ایچ پی وی ویکسین کے بارے میں ملنے والی معلومات پر بھروسہ کریں گے؟
27	Would you prefer to wait for efficacy of HPV vaccine being proven before deciding on the HPV vaccination?<br>کیا آپ ایچ پی وی ویکسین لگوانے سے پہلے یہ دیکھنا چاہیں گے کہ یہ واقعی مؤثر ہے

28	If a community leader recommends the HPV vaccine, would you be more willing to get the girls in your family vaccinated?<br>اگر آپ کی کمیونٹی کا کوئی ایسا شخص جس پر آپ بھروسہ کرتے ہیں ایچ پی وی ویکسین کی سفارش کرے تو کیا آپ اپنے خاندان کی لڑکیوں کو ویکسین لگوانے پر راضی ہو جائیں گے؟
29	Would your family involve the girls in discussions about HPV vaccination before deciding on vaccination?<br>کیا آپ کے خاندان والے ایچ پی وی ویکسین لگوانے کا فیصلہ کرنے سے پہلے لڑکیوں سے مشورہ کریں گے؟
30	Would you recommend the HPV vaccination to others?<br>کیا آپ دوسروں کو ایچ پی وی ویکسین لگوانے کا مشورہ دیں گے؟
31	Would you prefer that the healthcare provider educating about the HPV vaccine be a woman?<br>کیا آپ چاہیں گے کہ ایچ پی وی ویکسین کے بارے میں آگاہی دینے والا صحت کا کارکن خاتون ہو؟
32	To what extent do you trust the following sources for accurate information about the HPV vaccine?<br>آپ ایچ پی وی ویکسین سے متعلق درست معلومات حاصل کرنے کے لیے ایک صحت کے ماہر پر کتنا بھروسہ کرتے ہیں؟<br>آپ ایچ پی وی ویکسین سے متعلق درست معلومات حاصل کرنے کے لیے ایک کمیونٹی لیڈر پر کتنا بھروسہ کرتے ہیں؟<br>پ ایچ پی وی ویکسین سے متعلق درست معلومات حاصل کرنے کے لیے ایک مذہبی رہنما پر کتنا بھروسہ کرتے ہیں؟<br>آپ ایچ پی وی ویکسین سے متعلق درست معلومات حاصل کرنے کے لیے اپنے خاندان کے بزرگوں پر کتنا بھروسہ کرتے ہیں؟<br>آپ ایچ پی وی ویکسین سے متعلق درست معلومات حاصل کرنے کے لیے میڈیا پر کتنا بھروسہ کرتے ہیں؟
	**Domain 3: Motivation**
33	**Do you want your daughter to get vaccinated against HPV ?**<br>کیا آپ اپنی بیٹی کو ایچ پی وی ویکسین لگوانا چاہتے ہیں
34	When the HPV vaccine campaign begins, do you intend to register the girls in your family?<br>جب ایچ پی وی ویکسین کی مہم کا آغاز ہو گا، تو کیا آپ اپنے خاندان کی لڑکیوں کو رجسٹر کرانے کا ارادہ کریں گے؟
35	To what extent do you believe vaccinating girls against HPV is as important as receiving other childhood vaccines?<br>کیا آپ سمجھتے ہیں کے لڑکیوں کو ایچ پی وی ویکسین لگوانا دیگر بچوں کی ویکسینز جتنا ہی اہم ہے؟
36	If you know that cervical cancer cases are increasing among young women in your area, will it motivate you to get the girls in your family vaccinated?<br>گر آپ کو پتہ چلے کہ آپ کے علاقے میں نوجوان خواتین میں سرویکل کینسر کے کیسز بڑھ رہے ہیں تو کیا یہ معلومات آپ کو اپنے خاندان کی لڑکیوں کو ایچ پی وی ویکسین لگوانے پر آمادہ کرے گی ؟
37	Would your past experience with routine immunization impact your decision about HPV vaccination?<br>کیا معمول کی ویکسینیشن سے متعلق آپ کا سابقہ تجربہ ایچ پی وی ویکسین کے بارے میں آپ کے فیصلے پر اثر انداز ہوگا؟
38	Compared to other vaccines, how much do you trust the safety of the HPV vaccine?<br>دوسری ویکسینز کے مقابلے میں آپ کو ایچ پی وی ویکسین کے محفوظ ہونے پر کتنا بھروسہ ہے؟
39	If you are given scientific information about the HPV vaccine, would you consider getting the girls in your family vaccinated?

	اگر آپ کو ایچ پی وی ویکسین کے بارے میں سائنسی معلومات فراہم کی جائیں، □و کیا آپ اپنی فیملی کی لڑکیوں کو ویکسین لگو□ے پر غور کریں گے؟
40	To what extent would learning that other countries are administering the HPV vaccine increase your trust in receiving the vaccine?<br>اگر آپ کو پتہ □لے کہ دیگر ممالک میں ایچ پی وی ویکسین لگائی جا رہی ہے، □و کیا اس سے آپ کا ویکسین پر اعتماد بڑھے گا؟
	**Domain 4: Practical Issues**
**41**	**Have you ever been contacted about your daughter being due for HPV vaccination?**<br>کیا آپ کو کبھی اپنی بیٹی کو ایچ پی وی ویکسین لگو□ے کے لئے رجوع کیا گیا ہے
42	Would you get the girls in your family vaccinated if there is sufficient data available on the impact of usage of the HPV vaccine in Pakistan?<br>کیا آپ اپنی خا□دان کی لڑکیوں کو ایچ پی وی ویکسین لگوائیں گے اگر پاکستان میں اس کے استعمال کے اثرات سے متعلق کافی ڈیٹا موجود ہو؟
43	If healthcare facility staff provide sufficient information about the HPV vaccine, would it increase your confidence in choosing HPV vaccination?<br>اگر صحت کی سہولت کے عملے کی جا□ب سے ایچ پی وی ویکسین کے بارے میں کافی معلومات فراہم کی جائیں، □و کیا اس سے آپ کا اعتماد ایچ پی وی ویکسین لگو□ے کے فیصلے میں بڑھے گا؟
44	Do you think incentives such as financial rewards, free healthcare services, or educational benefits should be used to promote HPV vaccination?<br>کیا آپ سمجھتے ہیں کہ ایچ پی وی ویکسینیشن کو فروغ دینے کے لیے مالی □عامات، مفت صحت کی خدمات، یا □علیمی فوائد جیسے مراعات استعمال کی جا□ی □اہئیں؟
45	If HPV vaccination is made mandatory by the government, do you think schools are the appropriate places to vaccinate the girls?<br>اگر حکومت ایچ پی وی ویکسین کو لازمی قرار دے دے، □و کیا آپ سمجھتے ہیں کہ اسکول لڑکیوں کو ویکسین لگو□ے کے لیے مناسب جگہ ہیں؟
46	If HPV vaccination becomes a legal requirement (e.g., for school admission or travel), would you ensure that the girls in your family receive the vaccine?<br>اگر ایچ پی وی ویکسین کو قا□ون کے □حت لازمی قرار دیا جائے (جیسے اسکول میں داخلے یا سفر کے لیے)، □و کیا آپ اپنے خا□دان کی لڑکیوں کو یہ ویکسین لگو□ے کو یقینی بنائیں گے؟
47	Do you agree that public and private schools have different attitudes towards vaccination campaigns?<br>کیا آپ اس بات سے متفق ہیں کہ سرکاری اور □جی اسکول ویکسینیشن مہمات کے بارے میں مختلف رویّہ رکھتےہیں ؟
48	To what extent do you think teachers should receive specific training to raise awareness about the HPV vaccine among adolescent girls?<br>کیا آپ سمجھتے ہیں کہ اسا□ذہ کو □وجوان لڑکیوں میں ایچ پی وی ویکسین کی آگاہی پیدا کر□ے کے لئے الگ □ربیت دی جا□ی □اہیے ؟
49	Do you think girls should be given awareness about the HPV vaccine in schools to support successful launch?<br>کیا آپ سمجھتے ہیں کہ لڑکیوں کو اسکول میں ایچ پی وی ویکسین کے بارے میں آگاہی دی جا□ی □اہیے ؟
50	Do you trust outreach vaccination services for the HPV vaccination?<br>کیا آپ ایچ پی وی ویکسین لگو□ے کے لئے اوٹریچ ویکسینیشن سروسز پر بھروسہ کر□ے ہیں ؟
51	In your view, how much does it matter to people if the HPV vaccine is produced locally versus being imported?

	آپ کے خیال میں، لوگوں کے لئے یہ بات کتنی اہم ہے کہ ایچ پی وی ویکسین مقامی طور پر تیار کی گئی ہو یا باہر سے درآمد کی گئی ہو؟
52	Based on your past experience, to what extent do you think vaccination staff are cooperative?<br>آپ کے سابقہ تجربے کے مطابق، آپ کی رائے میں ویکسینیشن عملہ کس حد تک تعاون کرنے والا ہے؟
53	To what extent would you prefer each of the following places as the site for receiving the HPV vaccination? (School, Fixed site, outreach services)<br>آپ کس قدر مندرجہ زیل مقامات کو ایچ پی وی ویکسین لگوانے کے لئے مناسب سمجھتے ہیں۔اسکول ،ہسپتال ،آؤٹ ریچ سروسز
54	To what extent do you think each of the following barriers exists in your community regarding access to vaccines?<br>آپ کے خیال میں آپ کی کمیونٹی میں ویکسین تک رسائی میں کون سی رکاوٹیں ہو سکتی ہیں ؟<br>فاصلہ زیادہ ہونا، معلومات کی کمی، کام یا خاندانی ذمہ داریاں، ویکسین دستیاب نہ ہونا، ٹرانسپورٹ کی کمی، کلینک کے اوقات)
55	To what extent do you usually experience long waiting times at the health center for routine vaccination?<br>عام طور پر معمول کی ویکسینیشن کے لیے صحت کے مرکز میں آپ کو کتنی دیر انتظار کرنا پڑتا ہے؟
56	To what extent do you trust each of the following social media platforms for vaccine-related information? Platforms to be rated separately:<br>آپ ویکسین سے متعلق معلومات حاصل کرنے کے لیے کن سوشل میڈیا پلاٹف فارم پر سب سے زیادہ بھروسہ کرتے ہیں؟<br>ٹک ٹاک، فیس بک، انسٹاگرام، یوٹیوب، ٹوئٹر، دیگر)
57	To what extent do you think reminders (e.g., mobile messages, school announcements) are helpful in ensuring timely vaccination?<br>آپکے خیال میں بروقت ویکسینیشن کو یقینی بنانے کے لیے یاد دہانی کتنی مددگار ہوگی؟
58	To what extent do you receive vaccine-related information through TV, radio, or mobile phone?<br>آپ کو ٹی-وی، ریڈیو یا موبائل فون کے ذریعے ویکسین سے متعلق معلومات کتنی بار موصول ہوتی ہیں؟
	**Domain 5: Cultural Integration**
59	To what extent does each of the following usually make health decisions in your household? (Maternal grandparents, Paternal grandparents, Mother, Father, others)<br>مندرجہ زیل لوگ کس قدر آپ کے گھر میں فیصلہ سازی میں حصہ لیتے ہیں
60	Do you feel comfortable discussing women's health matters with your family?<br>کیا آپ اپنے اہل خانہ کے ساتھ خواتین کی صحت کے امور پر کھل کر اور بغیر ہچکچاہٹ کے بات کرنے میں آرام دہ محسوس کرتے ہیں؟
61	To what extent do you think men should be educated about the HPV vaccine through awareness campaigns?<br>کیا آپ سمجھتے ہیں کہ مردوں کو بھی ایچ پی وی ویکسین کے بارے میں آگاہی مہمات کے ذریعے تعلیم دی جانی چاہیے
62	To what extent would family opinions influence your decision about getting the girls in your family vaccinated?<br>آپ کے خاندان والوں کی رائے آپ کے گھر کی لڑکیوں کو ایچ پی وی ویکسینیشن کروانے کے فیصلے پر کس حد تک اثر انداز ہوگی ؟
63	If your daughter expresses her interest in receiving the HPV vaccine but you still have some concerns, how would you support this decision as a parent?

	اگر آپ کی بیٹی ایچ پی وی ویکسین لگوانے کی خواہش ظاہر کرے، لیکن آپ کو کچھ خدشات لاحق ہوں، تو کیا آپ والدین کی حیثیت سے اس کے فیصلے کی حمایت کریں گے
64	To what extent do you think that hearing real stories of cervical cancer patients would increase parents'/caregivers' trust in the HPV vaccine?<br>کیا آپ کے خیال میں سروائیکل کینسر کے مریضوں کی حقیقی کہانیاں سننے سے والدین کا ایچ پی وی ویکسین پر اعتماد بڑھ سکتا ہے؟
65	If you see health care professionals vaccinating their own daughters against HPV, would you feel more confident about doing the same?<br>اگر آپ دیکھیں کہ صحت کے ماہرین اپنی بیٹیوں کو ایچ پی وی ویکسین لگوا رہے ہیں، تو کیا آپ بھی ایسا کرنے میں زیادہ پُر اعتماد محسوس کریں گے؟
66	In your opinion, does the gender of the health worker affect acceptance of the HPV vaccine for women /girls ?<br>کیا آپ کے خیال میں، ویکسین لگانے والے صحت کے کارکن کی جنس کا لڑکیوں کی ایچ پی وی ویکسینیشن پر کوئی اثر پڑتا ہے؟
67	Do people in community hesitate to get the HPV vaccine due to social reasons?<br>کیا معاشرتی عوامل کی وجہ سے لوگ ایچ پی وی ویکسین لگوانے سے کتراتے ہیں؟
68	To what extent, do you think the gender focus of HPV vaccination programs affects how people view the vaccine?<br>کیا آپ کے نزدیک، ایچ پی وی ویکسینیشن پروگرام میں صنفی تفریق لوگوں کی ویکسین سے متعلق رائے کو متاثر کرسکتا ہے؟
69	Do you think cultural background influences families' willingness to vaccinate their daughters against HPV?<br>کیا آپ سمجھتے ہیں کہ ثقافتی پس منظر لوگوں کے ایچ پی وی ویکسینیشن کے انتخاب پر اثر انداز ہوتی ہے؟
70	How important is parental consent when offering the HPV vaccine to school-going girls?<br>اسکول کو لڑکیوں کو ایچ پی وی ویکسین لگانے کے لیے والدین کی رضا مندی حاصل کرنا کتنی ضروری ہے؟
	Vaccine Uptake Domain 6
71	Have you ever been contacted by a healthcare provider, school, or health program about your daughter being due for the HPV vaccine?کیا کبھی کسی صحت کے کارکن، اسکول، یا صحت کے پروگرام کی جانب سے آپ سے اس بارے میں رابطہ کیا گیا ہے کہ آپ کی بیٹی کو HPV ویکسین لگوانے کا وقت آ گیا ہے؟
72	Has your daughter received the HPV vaccine?کیا آپ کی بیٹی کو HPV ویکسین لگ چکی ہے؟
73	Do you plan to have your daughter vaccinated against HPV?کیا آپ مستقبل میں اپنی بیٹی کو HPV کے خلاف ویکسین لگوانے کا ارادہ رکھتے ہیں؟
74	How important do you think it is for your daughter to receive the HPV vaccine to protect her from cervical cancer and other HPV-related diseases?آپ کے خیال میں سروائیکل کینسر (رحم کے منہ کے کینسر) اور HPV سے متعلق دیگر بیماریوں سے بچاؤ کے لیے آپ کی بیٹی کو HPV ویکسین لگوانا کتنا اہم ہے؟
75	How easy would it be for you to arrange for your daughter to receive the HPV vaccine if you decided to vaccinate her?اگر آپ اپنی بیٹی کو HPV ویکسین لگوانے کا فیصلہ کریں، تو اس کے لیے ویکسین لگوانے کا انتظام کرنا آپ کے لیے کتنا آسان ہوگا؟
