# Supplementary material for: Development and Initial Validation of a Multidimensional Instrument for Assessing Determinants of HPV Vaccine Readiness and Uptake Among Caregivers in Pakistan: A PLS-SEM Approach
Source: Vaccines (Basel). 2026 Aug 31;14(9):761. doi: 10.3390/vaccines14090761 (PMC13611574; doi:10.3390/vaccines14090761)
Supplement: Supplementary file 1 [file vaccines-14-00761-s001.zip › vaccines-4419243-supplementary/Supplementary File S2.pdf]

# Item Reduction and Content Validity Table

# PLS-SEM Measurement Model – Preliminary to Final Model

# Construct 1: Thinking & Feeling

*Initial pool: 19 items (D1_1 – D1_19) | Retained: 5 | Removed: 14*

| Item Code | Preliminary Outer Loading | Status | Statistical Reason | Conceptual Consequence / Justification |
|---|---|---|---|---|
| D1_1 | 0.834 | **Removed** | Loading ≥ 0.70; meets threshold | Despite an acceptable loading, D1_1 exhibited high theoretical overlap with retained item D1_5, which captures the same affective-appraisal dimension with greater contextual specificity. Removing D1_1 achieves model parsimony; the emotional valence dimension it tapped is fully preserved by D1_5 and D1_9. Content coverage of the construct is not compromised. |
| D1_2 | 0.787 | **Removed** | Loading ≥ 0.70; meets threshold | D1_2 showed high theoretical redundancy with retained item D1_15. Both items probe cognitive evaluation tendencies, but D1_15 captures this facet with greater discriminant clarity. Removing D1_2 streamlines the scale without sacrificing breadth; the evaluative-cognition sub-dimension remains represented in the final model. |
| D1_3 | 0.820 | **Removed** | Loading ≥ 0.70; meets threshold | D1_3 demonstrated semantic overlap with D1_12, with which it shares a reflective-reasoning sub-facet. Conceptual ambiguity existed in that respondents may have interpreted D1_3 and D1_12 as tapping the same underlying thought pattern. D1_12 was preferred for its more precise wording. The reflective reasoning dimension is fully retained. |
| D1_4 | 0.885 | **Removed** | Loading ≥ 0.70; meets threshold | D1_4 had a high loading but substantial theoretical overlap with D1_14, the top-loading item for this construct. Both items address emotional intensity in health-related decision-making. D1_14 was retained |

| | | | | |
|---|---|---|---|---|
| | | | | as the stronger and more contextually anchored indicator; removing D1_4 achieves parsimony without sacrificing the emotional intensity sub-domain. |
| **D1_5** | **0.872** | **Retained** | Loading ≥ 0.70; strong indicator | D1_5 conceptually captures the affective-appraisal dimension of Thinking & Feeling, reflecting how respondents emotionally evaluate health-related information, which is central to the construct's theoretical definition. |
| D1_6 | 0.854 | **Removed** | Loading ≥ 0.70; meets threshold | D1_6 displayed high theoretical overlap with D1_9 and D1_14, both of which are retained. Conceptually, D1_6 and D1_9 tap the same reflexive-feeling sub-dimension, making D1_6 redundant. Removing D1_6 does not reduce coverage of the feeling-based processing facet, which is adequately represented by D1_9 and D1_15. |
| D1_7 | 0.893 | **Removed** | Loading ≥ 0.70; meets threshold | Although D1_7 carries an excellent loading, it is conceptually redundant with D1_9 (loading = 0.888), with both items addressing the deliberative cognitive component of the construct. D1_9 was selected as it more directly references the decision-making context. Removing D1_7 preserves model parsimony without any loss of sub-domain coverage. |
| D1_8 | 0.869 | **Removed** | Loading ≥ 0.70; meets threshold | D1_8 exhibited conceptual ambiguity—respondents may have interpreted it as overlapping with both the Thinking and the Social Processes constructs, given its wording around interpersonally influenced affect. This cross-construct ambiguity, combined with its redundancy with D1_9, justified its removal. The emotionally-driven deliberation facet is captured by D1_9 and D1_14. |
| **D1_9** | **0.888** | **Retained** | Loading ≥ 0.70; strong indicator | D1_9 anchors the deliberative cognitive dimension of the construct, capturing how individuals consciously process and reflect on health-related feelings, a core facet of the Thinking & Feeling domain. |

| | | | | |
|---|---|---|---|---|
| D1_10 | 0.839 | **Removed** | Loading ≥ 0.70; meets threshold | D1_10 showed semantic redundancy with D1_12, as both address memory-based or recall-driven emotional processing. D1_12 was retained on the basis of superior conceptual precision. Removal of D1_10 does not diminish the recall-emotion sub-dimension, which remains represented in the final model. |
| D1_11 | 0.770 | **Removed** | Loading ≥ 0.70; meets threshold | D1_11 meets the threshold but functions as a near-duplicate of D1_15 at the conceptual level, both addressing mild or moderate affective responses to health messaging. D1_15 was retained as the stronger indicator. Removing D1_11 achieves parsimony; the mild-affect sub-domain is preserved. |
| **D1_12** | **0.834** | **Retained** | Loading ≥ 0.70; strong indicator | D1_12 captures the reflective-reasoning sub-dimension of Thinking & Feeling—the cognitive effort invested when processing vaccination-related information. This facet is theoretically distinct from pure emotion and is essential to the construct's definitional breadth. |
| D1_13 | 0.907 | **Removed** | Loading ≥ 0.70; meets threshold | D1_13 is the single highest-loading item in this pool yet was removed for conceptual reasons: its wording was judged to have high theoretical overlap with D1_14, and the two items together would introduce multicollinearity risk. D1_14 was selected over D1_13 because it captures the emotional response dimension with greater precision relative to the construct's nomological framework. The apex-emotional facet is fully retained through D1_14. |
| **D1_14** | **0.900** | **Retained** | Loading ≥ 0.70; strong indicator | D1_14 anchors the emotional intensity dimension, reflecting strong affective reactions to vaccination decisions—the core evaluative-feeling nucleus of the construct as theoretically defined. |
| **D1_15** | **0.799** | **Retained** | Loading ≥ 0.70; strong indicator | D1_15 captures mild-to-moderate affective processing, ensuring that the full range of emotional intensity within the construct's domain is represented, from low-valence |

| | | | | |
|---|---|---|---|---|
| | | | | evaluation through to the high-intensity response captured by D1_14. |
| D1_16 | −0.025 | **Removed** | Loading near zero / below threshold | D1_16 produced a near-zero and negative loading, indicating no reliable variance shared with the Thinking & Feeling latent construct. Conceptually, item wording was too broadly framed, lacking the contextual specificity required to activate the cognitive-affective processes central to this construct. Removing it eliminates measurement noise without any loss of construct coverage. |
| D1_17 | 0.015 | **Removed** | Loading near zero / below threshold | D1_17 demonstrated a negligible loading, signalling conceptual misalignment with the construct. The item was phrased in abstract terms that failed to trigger the specific health-decision emotional responses the construct seeks to measure. Its removal has no consequence for domain coverage. |
| D1_18 | −0.023 | **Removed** | Loading near zero / below threshold | D1_18 produced a near-zero loading and conceptually appeared to tap situational affect rather than the durable emotional-cognitive orientations that define Thinking & Feeling. Removing it does not affect construct coverage. |
| D1_19 | −0.061 | **Removed** | Loading below threshold; negative | D1_19 showed a small negative loading, suggesting it may be reverse-functioning or measuring a peripheral aspect unrelated to the construct's core. Its item wording was too broad and lacked the contextual specificity needed. Removal eliminates measurement error and does not compromise domain coverage. |

---

# Construct 2: Social Processes

*Initial pool: 13 items (D2_1 – D2_13) | Retained: 6 | Removed: 7*

| Item Code | Preliminary Outer Loading | Status | Statistical Reason | Conceptual Consequence / Justification |
|---|---|---|---|---|
| **D2_1** | **0.794** | **Retained** | Loading ≥ 0.70; strong indicator | D2_1 captures the interpersonal influence dimension of Social Processes—specifically, the role of social norms and peer expectations in shaping vaccination attitudes—which is foundational to the construct's theoretical definition. |
| **D2_2** | **0.856** | **Retained** | Loading ≥ 0.70; strong indicator | D2_2 anchors the social conformity sub-dimension, reflecting how reference group expectations shape health behaviour intentions, a core component of social-process theories of health behaviour. |
| **D2_3** | **0.868** | **Retained** | Loading ≥ 0.70; strong indicator | D2_3 captures the information-sharing and social learning facet—how individuals acquire vaccination-related beliefs through social networks—constituting a theoretically distinct and indispensable dimension of Social Processes. |
| **D2_4** | **0.863** | **Retained** | Loading ≥ 0.70; strong indicator | D2_4 reflects the trust-in-community dimension, representing how confidence in communal norms and collective behaviour motivates individual health decisions, a construct-defining facet that cannot be omitted without conceptual loss. |
| **D2_5** | **0.894** | **Retained** | Loading ≥ 0.70; strong indicator | D2_5 is the strongest indicator of Social Processes, anchoring the social endorsement sub-dimension—the explicit approval or encouragement received from significant others—and captures a core definitional element of the construct. |
| **D2_6** | **0.813** | **Retained** | Loading ≥ 0.70; strong indicator | D2_6 captures the social accountability dimension, reflecting obligations arising from group membership and communal identity, rounding out the Social Processes construct and ensuring full domain coverage. |
| D2_7 | −0.020 | **Removed** | Loading near zero / below threshold | D2_7 returned a near-zero loading, indicating conceptual misfit with the Social Processes construct. Its wording was too individualistic, failing to tap the relational dynamics the construct is defined by. Removal has no consequence for domain coverage. |
| D2_8 | −0.046 | **Removed** | Loading near zero / below threshold | D2_8 demonstrated a near-zero negative loading. The item wording was too broad and lacked contextual specificity—it could be interpreted as referencing personal deliberation rather than social influence |

| Item Code | Preliminary Outer Loading | Status | Statistical Reason | Conceptual Consequence / Justification |
|---|---|---|---|---|
| | | | | processes. Removing it does not compromise construct domain coverage. |
| D2_9 | −0.033 | **Removed** | Loading near zero / below threshold | D2_9 showed a negligible loading. Conceptually, the item wording overlapped with general attitudinal content rather than targeting the social-relational mechanisms specific to this construct. Its removal eliminates irrelevant variance without affecting coverage. |
| D2_10 | −0.088 | **Removed** | Loading below threshold; negative | D2_10 produced a negative loading, suggesting it may measure individual resistance rather than social facilitation. Its wording lacked the positive social-dynamic orientation that defines Social Processes in this model. Removing it removes measurement noise; the six retained items fully represent the construct's theoretical domain. |
| D2_11 | −0.012 | **Removed** | Loading near zero / below threshold | D2_11 had a negligible loading and was conceptually extraneous—its item content addressed administrative or systemic processes rather than interpersonal social dynamics. Removing it does not compromise construct breadth. |
| D2_12 | 0.029 | **Removed** | Loading below threshold | D2_12 exhibited a trivially small loading. Conceptually, the item wording was too broad and failed to activate the interpersonal social processes the construct represents. The social influence domain is fully covered by the six retained items. |
| D2_13 | 0.034 | **Removed** | Loading below threshold | D2_13 had a negligible loading. Semantic overlap existed with peripheral aspects of the Practical Issues construct rather than the social-relational core of Social Processes. Removing it does not diminish the social-process domain, which is comprehensively captured by D2_1 through D2_6. |

## Construct 3: Motivation

*Initial pool: 8 items (D3_1 – D3_8) | Retained: 6 | Removed: 2*

| Item Code | Preliminary Outer Loading | Status | Statistical Reason | Conceptual Consequence / Justification |
|---|---|---|---|---|
| **D3_1** | **0.837** | **Retained** | Loading ≥ 0.70; strong indicator | D3_1 anchors the intrinsic motivation dimension—personal desire to protect one's own health—which is a foundational sub-component of vaccination motivation as theoretically defined. |
| **D3_2** | **0.838** | **Retained** | Loading ≥ 0.70; strong indicator | D3_2 captures protective-other motivation, reflecting the drive to vaccinate in order to protect family or community members—a distinct and theoretically essential dimension of the Motivation construct. |
| **D3_3** | **0.796** | **Retained** | Loading ≥ 0.70; strong indicator | D3_3 represents the preventive health orientation dimension, capturing goal-directed motivation linked to disease avoidance, which is conceptually non-redundant with the affective or social motivational facets covered by other retained items. |
| **D3_4** | **0.845** | **Retained** | Loading ≥ 0.70; strong indicator | D3_4 captures efficacy-based motivation—confidence that vaccination will be effective—which is a theoretically distinct sub-dimension in established health motivation frameworks and essential to the construct's definitional completeness. |
| **D3_5** | **0.892** | **Retained** | Loading ≥ 0.70; strongest indicator | D3_5 is the strongest indicator of the Motivation construct. It captures comprehensive health-seeking drive, integrating both self-protective and community-protective motivational orientations, making it the construct's anchor item. |
| **D3_6** | **0.838** | ✓ **Retained** | Loading ≥ 0.70; strong indicator | D3_6 captures the normative-compliance motivation dimension—being motivated by recommendations from health authorities—which represents a theoretically distinct facet that ensures the retained indicator set covers the full motivational spectrum. |
| D3_7 | 0.134 | **Removed** | Loading well below threshold (< 0.70) | D3_7 showed a weak loading, indicating that its item content does not reliably represent the Motivation construct. Conceptually, the item wording was too abstract and context-independent, failing to activate the specific vaccination-related motivational processes the construct targets. Removing D3_7 does not compromise domain coverage; all major motivational sub-facets—intrinsic, altruistic, |

| Item Code | Preliminary Outer Loading | Status | Statistical Reason | Conceptual Consequence / Justification |
|---|---|---|---|---|
| | | | | preventive, efficacy-based, and normative—are represented by the six retained items. |
| D3_8 | 0.080 | **Removed** | Loading well below threshold (< 0.70) | D3_8 exhibited a negligible loading. Conceptually, the item appeared to address situational or external motivators tangential to the stable, enduring motivational orientations that theoretically define this construct. Its removal eliminates measurement noise; the six retained items comprehensively capture the construct's domain. |

# Construct 4: Practical Issues

*Initial pool: 18 items (D4_1 – D4_18) | Retained: 3 | Removed: 15*

| Item Code | Preliminary Outer Loading | Status | Statistical Reason | Conceptual Consequence / Justification |
|---|---|---|---|---|
| **D4_1** | **0.908** | **Retained** | Loading ≥ 0.70; strong indicator | D4_1 captures the accessibility dimension of Practical Issues—physical and logistical access to vaccination services—which is a foundational sub-domain of structural barriers to vaccination uptake. |
| **D4_2** | **0.898** | **Retained** | Loading ≥ 0.70; strong indicator | D4_2 anchors the time and scheduling constraints dimension, reflecting how competing demands on time create practical barriers, a theoretically distinct and non-redundant facet of the construct. |
| **D4_3** | **0.917** | **Retained** | Loading ≥ 0.70; strong indicator | D4_3 captures the cost and financial barrier dimension, representing economic obstacles to vaccination—a conceptually distinct sub-domain that ensures the retained items collectively represent the full practical-barrier landscape. |
| D4_4 | 0.948 | **Removed** | Loading ≥ 0.70; highest in pool | Despite having the highest loading in the entire construct pool, D4_4 was removed on conceptual grounds. Substantive theoretical overlap exists between D4_4 and D4_3: both address resource-related barriers to vaccine access. Given the near-perfect redundancy in construct coverage and the risk of multicollinearity, D4_3 was retained as the more contextually precise indicator. Removing D4_4 achieves parsimony; the |

| Item Code | Preliminary Outer Loading | Status | Statistical Reason | Conceptual Consequence / Justification |
|---|---|---|---|---|
| | | | | resource-barrier dimension is fully preserved by D4_3. |
| D4_5 | 0.885 | **Removed** | Loading ≥ 0.70; meets threshold | D4_5 demonstrated high conceptual overlap with D4_1, with both items tapping the physical accessibility dimension. D4_1 was retained as the more theoretically precise indicator of geographic and logistical access. Removing D4_5 achieves model parsimony without sacrificing construct coverage; the accessibility facet is fully represented by D4_1. |
| D4_6 | −0.077 | **Removed** | Loading below threshold; negative | D4_6 produced a negative and near-zero loading, signalling conceptual misfit. The item wording was too vague and lacked specificity to the structural, logistical barriers that define Practical Issues. Removing it eliminates measurement noise; no domain coverage is lost. |
| D4_7 | −0.073 | **Removed** | Loading below threshold; negative | D4_7 showed a small negative loading. Conceptually, the item wording was too broad—it could be interpreted as addressing attitudinal rather than practical barriers—resulting in conceptual ambiguity and misalignment with the construct. Its removal has no consequence for domain coverage. |
| D4_8 | −0.005 | **Removed** | Loading near zero / below threshold | D4_8 produced a negligible loading. The item wording lacked contextual specificity and did not reliably discriminate practical from other barrier types. Removing it does not affect construct coverage. |
| D4_9 | −0.005 | **Removed** | Loading near zero / below threshold | D4_9 displayed a trivially small loading. Conceptually, its wording was too abstract and did not target the concrete, actionable practical obstacles the construct is intended to measure. No domain coverage is lost through its removal. |
| D4_10 | 0.086 | **Removed** | Loading well below threshold (< 0.70) | D4_10 showed a weak loading, indicating insufficient alignment with the Practical Issues construct. Its item content addressed peripheral logistical considerations that are not core to the construct's definitional scope. Removing it does not compromise construct coverage. |
| D4_11 | −0.174 | **Removed** | Loading below | D4_11 produced a negative loading, suggesting it may be functioning in the |

| Item Code | Preliminary Outer Loading | Status | Statistical Reason | Conceptual Consequence / Justification |
|---|---|---|---|---|
| | | | threshold; negative | opposite direction from the construct. Conceptually, the item appeared to tap perceived barriers in a way that conflates personal agency with structural obstacles, introducing construct-level ambiguity. Its removal eliminates systematic measurement error. |
| D4_12 | 0.048 | **Removed** | Loading well below threshold (< 0.70) | D4_12 exhibited a near-zero loading. Semantic redundancy existed with general practical-difficulty language, but the item lacked the construct-specific framing needed to anchor it to vaccination-related structural barriers. Removing it has no consequence for domain coverage. |
| D4_13 | −0.073 | **Removed** | Loading below threshold; negative | D4_13 showed a small negative loading. Its wording was too broadly framed and lacked contextual specificity, failing to activate responses about concrete structural impediments to vaccination. Removing it does not compromise the construct's theoretical scope. |
| D4_14 | −0.029 | **Removed** | Loading near zero / below threshold | D4_14 produced a negligible loading. Conceptually, the item wording overlapped with attitudinal content rather than the practical, situational barriers the Practical Issues construct is designed to capture. Its removal eliminates noise without affecting coverage. |
| D4_15 | 0.008 | **Removed** | Loading near zero / below threshold | D4_15 showed a negligible loading. The item was worded too broadly, lacking the specificity required to reliably reflect the structural barriers to vaccination uptake central to this construct. No domain coverage is lost. |
| D4_16 | 0.172 | **Removed** | Loading well below threshold (< 0.70) | D4_16 demonstrated a weak loading. Conceptually, its wording was too general, addressing practical inconvenience in the abstract rather than the specific logistical, cost, and time barriers that constitute the Practical Issues construct. Removing it does not diminish the construct's theoretical coverage. |
| D4_17 | 0.053 | **Removed** | Loading well below | D4_17 showed a negligible loading. Semantic redundancy existed with D4_2 at a surface level, but D4_17 lacked the precision |

| Item Code | Preliminary Outer Loading | Status | Statistical Reason | Conceptual Consequence / Justification |
| --- | --- | --- | --- | --- |
| | | | threshold (< 0.70) | required to serve as a valid indicator. Its removal is conceptually harmless; the scheduling-barrier facet is fully captured by D4_2. |
| D4_18 | 0.051 | **Removed** | Loading well below threshold (< 0.70) | D4_18 produced a near-zero loading. Its item content was peripheral to the core definitional space of Practical Issues and showed semantic redundancy with general logistical language rather than construct-specific content. Removing it eliminates measurement noise; no coverage is lost. |

---

# Construct 5: Cultural Integration

*Initial pool: 12 items (D5_1 – D5_12) | Retained: 3 | Removed: 9*

| Item Code | Preliminary Outer Loading | Status | Statistical Reason | Conceptual Consequence / Justification |
| --- | --- | --- | --- | --- |
| **D5_1** | **0.794** | **Retained** | Loading ≥ 0.70; strong indicator | D5_1 captures the cultural identity alignment dimension—how individuals' cultural values and identity are consistent with vaccination norms—which is theoretically foundational to the construct of Cultural Integration and cannot be omitted without conceptual loss. |
| D5_2 | 0.906 | **Removed** | Loading ≥ 0.70; strong loading | Despite its high loading, D5_2 was removed due to high theoretical overlap with D5_4, the retained anchor item. Both items address the degree of integration of health norms into cultural practice, but D5_4 does so with greater specificity and discriminant clarity relative to the broader nomological network. Removing D5_2 achieves parsimony while preserving the cultural-norm integration facet via D5_4. |
| **D5_3** | **0.873** | **Retained** | Loading ≥ 0.70; strong indicator | D5_3 captures the cultural tradition and ritual dimension—the extent to which health-protective practices align with or are embedded in cultural traditions—representing a theoretically distinct sub-domain of Cultural Integration that is essential to the construct's definitional completeness. |

| Item Code | Preliminary Outer Loading | Status | Statistical Reason | Conceptual Consequence / Justification |
|---|---|---|---|---|
| **D5_4** | **0.910** | **Retained** | Loading ≥ 0.70; strongest indicator | D5_4 is the strongest retained indicator, anchoring the cultural value congruence dimension—the alignment between personal cultural values and the perceived value of vaccination. It is the theoretical centrepiece of the Cultural Integration construct. |
| D5_5 | 0.128 | **Removed** | Loading well below threshold (< 0.70) | D5_5 showed a weak loading, indicating poor alignment with the Cultural Integration latent factor. Item wording was too broad and lacked contextual specificity, failing to differentiate cultural integration from general social conformity. Its removal does not compromise construct coverage. |
| D5_6 | 0.087 | **Removed** | Loading well below threshold (< 0.70) | D5_6 exhibited a near-zero loading. Conceptually, the item addressed peripheral cultural preferences rather than the core value-health alignment processes the construct targets. Removing it eliminates irrelevant variance without affecting domain coverage. |
| D5_7 | 0.152 | **Removed** | Loading well below threshold (< 0.70) | D5_7 showed a weak loading. Its item wording was too abstract and lacked the vaccine-specific cultural context required to function as a valid indicator of Cultural Integration. Removing it has no consequence for construct coverage. |
| D5_8 | 0.182 | **Removed** | Loading well below threshold (< 0.70) | D5_8 produced a weak loading. Semantic redundancy existed with D5_1 at a general cultural-values level, but D5_8 lacked the definitional precision to serve as a distinct indicator. The cultural identity dimension is fully captured by D5_1. Its removal does not compromise domain coverage. |
| D5_9 | 0.192 | **Removed** | Loading well below threshold (< 0.70) | D5_9 showed a weak loading. Item wording was too broadly framed around cultural exposure rather than the active integration of cultural values with health behaviours that the construct represents. Removing it does not affect theoretical coverage. |
| D5_10 | 0.193 | **Removed** | Loading well below threshold (< 0.70) | D5_10 demonstrated a weak loading. Conceptually, its content addressed acculturation-adjacent processes that, while related, fall outside the specific cultural value-health alignment domain of this |

| Item Code | Preliminary Outer Loading | Status | Statistical Reason | Conceptual Consequence / Justification |
|---|---|---|---|---|
| | | | | construct. Removing it does not diminish the construct's definitional scope. |
| D5_11 | 0.043 | **Removed** | Loading near zero / below threshold | D5_11 produced a negligible loading. The item wording was too general and lacked construct-specific cultural context, resulting in near-zero shared variance with the Cultural Integration factor. Its removal eliminates noise; no domain coverage is affected. |
| D5_12 | 0.175 | **Removed** | Loading well below threshold (< 0.70) | D5_12 showed a weak loading. Semantic redundancy existed with D5_3 at the level of cultural tradition, but D5_12 lacked the specificity to discriminate cultural tradition effects on vaccination from broader lifestyle orientations. D5_3 is retained as the more precise indicator; removing D5_12 does not affect coverage. |

---

# Construct 6: Vaccination Uptake

*Initial pool: 5 items (D6_1 – D6_5) | Retained: 4 | Removed: 1*

| Item Code | Preliminary Outer Loading | Status | Statistical Reason | Conceptual Consequence / Justification |
|---|---|---|---|---|
| D6_1 | 0.698 | **Removed** | Loading below 0.70 threshold | D6_1 marginally failed the 0.70 threshold. Conceptually, its item wording was too broadly framed around general health-seeking intention rather than specifying vaccination uptake behaviour per se, introducing semantic ambiguity regarding the precise outcome being measured. This conceptual imprecision, combined with the sub-threshold loading, made its removal appropriate. The behavioural intention facet it partially addressed is more precisely captured by D6_2, and the act of uptake itself is represented by D6_3–D6_5. No definitional gap is created. |
| **D6_2** | **0.848** | **Retained** | Loading ≥ 0.70; strong indicator | D6_2 captures the behavioural intention dimension of Vaccination Uptake—the expressed intention to seek and receive a vaccine—which is a foundational precursor to |

| Item Code | Preliminary Outer Loading | Status | Statistical Reason | Conceptual Consequence / Justification |
|---|---|---|---|---|
| | | | | actual uptake behaviour and theoretically integral to the construct. |
| **D6_3** | **0.883** | **Retained** | Loading ≥ 0.70; strongest indicator | D6_3 is the strongest indicator, anchoring the actual vaccination completion dimension—whether the individual has received the vaccine—the most direct behavioural manifestation of the construct and its theoretical core. |
| **D6_4** | **0.862** | **Retained** | Loading ≥ 0.70; strong indicator | D6_4 captures the repeat/booster uptake dimension, reflecting sustained vaccination behaviour over time, which is a theoretically distinct and important facet of the Vaccination Uptake construct's full definitional scope. |
| **D6_5** | **0.860** | **Retained** | Loading ≥ 0.70; strong indicator | D6_5 captures the future vaccine acceptance dimension—willingness to receive future doses or related vaccines—ensuring the construct represents not only past and current uptake but also prospective vaccination behaviour, achieving full temporal coverage. |

# Summary Statistics

| Latent Construct | Total Items | Retained | Removed | Retained Items |
|---|---|---|---|---|
| Thinking & Feeling | 19 | 5 | 14 | D1_5, D1_9, D1_12, D1_14, D1_15 |
| Social Processes | 13 | 6 | 7 | D2_1–D2_6 |
| Motivation | 8 | 6 | 2 | D3_1–D3_6 |
| Practical Issues | 18 | 3 | 15 | D4_1, D4_2, D4_3 |
| Cultural Integration | 12 | 3 | 9 | D5_1, D5_3, D5_4 |
| Vaccination Uptake | 5 | 4 | 1 | D6_2–D6_5 |
| **Total** | **75** | **27** | **48** | |
