# Supplementary material for: Development and Initial Validation of a Multidimensional Instrument for Assessing Determinants of HPV Vaccine Readiness and Uptake Among Caregivers in Pakistan: A PLS-SEM Approach
Source: Vaccines (Basel). 2026 Aug 31;14(9):761. doi: 10.3390/vaccines14090761 (PMC13611574; doi:10.3390/vaccines14090761)
Supplement: Supplementary file 1 [file vaccines-14-00761-s001.zip › vaccines-4419243-supplementary/Supplementary File S3.pdf]

Modified Survey after Analysis (27 Item)

Code	Variable	Code	Statements
1	Thinking and Feeling	D1_5	In your opinion, do vaccines contain ingredients that are prohibited in religion (pork or alcohol)?
2		D1_9	How much do you trust the HPV vaccine being introduced in your country?
3		D1_12	Do you believe the HPV vaccine is acceptable in a conservative society?
4		D1_14	If you think the women in your family are at low risk of cervical cancer due to family history, would you still vaccinate them against HPV?
5		D1_15	Do you think the HPV vaccine will help protect women in your family from serious illness?
6	Social Processes	D2_1	Do you think most parents you know would want you to get your daughter vaccinated against HPV ?
7		D2_2	Do you think most of your close family and friends want you to get your daughter vaccinated against HPV?
8		D2_3	Has a health worker recommended your daughter vaccinated against HPV?
9		D2_4	If it was time for your daughter to get vaccinated, would the mother need permission to take your child to the clinic?
10		D2_5	How often do you hear rumors or misconceptions about the HPV vaccine in your community?
11		D2_6	To what extent do religious beliefs in your community influence decisions about the HPV vaccine?
12	Motivation	D3_1	Do you want your daughter to get vaccinated against HPV ?
13		D3_2	When the HPV vaccine campaign begins, do you intend to register the girls in your family?
14		D3_3	To what extent do you believe vaccinating girls against HPV is as important as receiving other childhood vaccines?
15		D3_4	If you know that cervical cancer cases are increasing among young women in your area, will it motivate you to get the girls in your family vaccinated?
16		D3_5	Would your past experience with routine immunization impact your decision about HPV vaccination?
17		D3_6	Compared to other vaccines, how much do you trust the safety of the HPV vaccine?
18	Practical Issues	D4_1	Have you ever been contacted about your daughter being due for HPV vaccination?
19		D4_2	Would you get the girls in your family vaccinated if there is sufficient data available on the impact of usage of the HPV vaccine in Pakistan?
20		D4_3	If healthcare facility staff provide sufficient information about the HPV vaccine, would it increase your confidence in choosing HPV vaccination?

21	Cultural Integration	D5_1	To what extent does each of the following usually make health decisions in your household? (Maternal grandparents, Paternal grandparents, Mother, Father, others)
22		D5_3	To what extent do you think men should be educated about the HPV vaccine through awareness campaigns?
23		D5_4	To what extent would family opinions influence your decision about getting the girls in your family vaccinated?
24	Vaccination Uptake	D6_2	Has your daughter received the HPV vaccine?
25		D6_3	Do you plan to have your daughter vaccinated against HPV?
26		D6_4	How important do you think it is for your daughter to receive the HPV vaccine to protect her from cervical cancer and other HPV-related diseases?
27		D6_5	How easy would it be for you to arrange for your daughter to receive the HPV vaccine if you decided to vaccinate her?
